# Comparative transcriptomics identifies genes underlying growth performance of the Pacific black-lipped pearl oyster *Pinctada margaritifera*

**DOI:** 10.1186/s12864-024-10636-0

**Published:** 2024-07-24

**Authors:** Y. Dorant, V. Quillien, J. Le Luyer, C. L. Ky

**Affiliations:** 1 Ifremer, ILM, IRD, UPF, UMR 241 SECOPOL, Polynésie française, Taravao, Tahiti, France; 2 IHPE, UMR 5244, Université de Montpellier, CNRS, Université de Perpignan Via Domitia, Ifremer, Montpellier, France; 3 Ifremer, Univ Brest, CNRS, IRD, UMR 6539, LEMAR, Plouzane, F-29280 France

**Keywords:** Growth, Bivalves, *Pinctada margaritifera*, RNA-seq, Oysters

## Abstract

**Background:**

In bivalves, the rate at which organisms grow is a major functional trait underlying many aspects of their commercial production. Growth is a highly polygenic trait, which is typically regulated by many genes with small to moderate effects. Due to its complexity, growth variability in such shellfish remains poorly understood. In this study, we aimed to investigate differential gene expression among spat of the pearl oyster *Pinctada margaritifera* with distinct growth phenotypes.

**Results:**

We selected two groups of *P. margaritifera* spat belonging to the same F2 cohort based on their growth performance at 5.5 months old. Transcriptome profile analysis identified a total of 394 differentially expressed genes between these Fast-growing (F) and Slow-growing (S) phenotypes. According to functional enrichment analysis, S oysters overexpressed genes associated with stress-pathways and regulation of innate immune responses. In contrast, F oysters up-regulated genes associated with cytoskeleton activity, cell proliferation, and apoptosis. Analysis of genome polymorphism identified 16 single nucleotide polymorphisms (SNPs) significantly associated with the growth phenotypes. SNP effect categorization revealed one SNP identified for high effect and annotated for a stop codon gained mutation. Interestingly, this SNP is located within a gene annotated for scavenger receptor class F member 1 (SRF1), which is known to modulate apoptosis. Our analyses also revealed that all F oysters showed up-regulation for this gene and were homozygous for the stop-codon mutation. Conversely, S oysters had a heterozygous genotype and a reduced expression of this gene.

**Conclusions:**

Altogether, our findings suggest that differences in growth among the same oyster cohort may be explained by contrasted metabolic allocation between regulatory pathways for growth and the immune system. This study provides a valuable contribution towards our understanding of the molecular components associated with growth performance in the pearl oyster *P. margaritifera* and bivalves in general.

**Supplementary Information:**

The online version contains supplementary material available at 10.1186/s12864-024-10636-0.

## Introduction

### Relevance and control of growth phenotype expression

Heterogeneity of body sizes is a common feature in wild and farmed populations belonging to the same cohort. In aquaculture, inter-individual variability is a major drawback for productivity and profitability; hence substantial efforts (mainly zootechnical) have been made to reduce this heterogeneity, while improving mean growth rate [1, 2]. Growth potential is one of the principal traits targeted in selective breeding programs [3, 4], yet the genomic architecture conditioning this potential remains poorly understood in shellfish, particularly bivalve species.

### Factors controlling growth rate and relation to other phenotypic traits

In aquaculture as in nature, growth is a complex trait under control of genetics and the environment, including both biotic and abiotic factors [1, 5, 6]. For instance, several studies have sought to quantify the effects on growth rate of different exogenous factors, such as nutritional manipulation [7–9] or temperature [10]. In parallel, controlled-environment studies comparing Slow- and Fast-growing phenotypes reported substantial basal physiological differences, suggesting that variance in growth rate is also intrinsically regulated by endogenous mechanisms [11, 12]. Quantitative genetics studies show that heritability of growth in bivalves is medium to high depending on the species (ranging 0.18 to 0.50), and thus sufficient for significant genetic gain [13–16]. However, higher growth rate has frequently been associated with reduced immune capacity or increased potential for pathogens and/or parasite development [17, 18]; hence, there is a need to understand the biological processes behind growth traits and associated phenotypes.

### Mechanisms – the genomic architecture of growth rate

The emergence of high-throughput sequencing technologies, notably whole transcriptome sequencing and genotyping by sequencing approaches, have significantly improved our ability to explore the molecular architectures of phenotype determination, including for non-model shellfish species. On the one hand, development of SNP markers allows detection of regions (quantitative trait loci, QTLs), specific candidate genes (RNAseq), or common genetic variants associated with a trait of interest. Indeed, quantitative genetic analyses also widely demonstrate that genome polymorphisms (e.g., SNPs) influence gene expression variation and downstream phenotypes, including their growth traits and performance [19]. Such genomic influence on growth traits has been repeatedly demonstrated in many bivalve species [20–25]. Similarly, genome-wide association study (GWAS) experiments revealed the polygenic nature of growth rate determination in Pacific oyster *Crassostrea gigas* [15, 26, 27]. On the other hand, transcriptomic approaches conducted in shellfish species have revealed differential gene expression and biological functions associated with differential growth, immunity, reproduction and biomineralization [28–37]. For instance, up-regulation of many genes involved in cellular control modulating cell proliferation, cell differentiation or cell death was observed between fast- and slow-growing phenotypes in the carpet shell clam *Ruditapes decussatus* [33], Manila clam *Ruditapes philippinarum* [38], and Pacific oyster *C. gigas* [39]. Lastly, a few studies have coupled genetic variant surveys and genes expression analysis to further trace phenotypic variation at the molecular levels. For example, SNPs significantly associated with growth differences in *C. gigas* have been attributed to functional genes such as those for amylase [40, 41] and insulin-related peptide [42]. Moreover, faster growth performance has been repeatedly correlated with heterozygous individuals, which were also characterized by raised metabolisms and protein turnovers [43]. Similarly, expression quantitative trait loci (eQTL) also offer an elegant approach associating genetics and genomics to identify underlying genetic architecture responsible for phenotypic variation in growth traits [37, 44]. For example, a causal SNP has been identified within the promotor region of the myostatin gene, which was significantly associated with higher growth trait values of heterozygous individuals of the clam species *Chlamy nobilis* [44]. Correlation analyses between genetic polymorphism and myostatin gene expression for growth traits have also been investigated in the scallop *Argopecten irradians* [45]. In this latter case, the authors identified two SNPs that together formed a causal haplotype associated with growth performance. Interestingly, they also found that heterozygous genotypes are not always the strongest drivers for fast growing phenotypes and that homozygous genotypes may also be important in explaining higher body mass. Overall, the availability of gene/genome information combined with transcriptomic data can be considered a key step to studying putative correlations between genome polymorphism and gene expression levels pertaining to downstream phenotypes of interest such as growth traits.

### Model species

The black-lipped pearl oyster (*P. margaritifera*) is the primary aquaculture species in French Polynesia and represents the second largest source of economic income after tourism for its cultured pearl production [46]. In such aquaculture, the body size of oysters represents an important phenotypic trait for farmers as it plays a role in many aspects of production such as animal stocking, predation and grafting [47]. On the one hand, greater oyster shell size (for individuals < 3 years old), especially for the recipient oyster in the grafting procedure, is accompanied by positive correlation with the size of the harvested pearls [48, 49]. Although the value of a cultured pearl mainly depends on surface quality traits (e.g., luster, shape, color), pearl size and weight are important factors that can substantially increase the value of a pearl, as the price is correlated with the weight. On the other hand, growth performance can also represent a critical trait for donor selection. Indeed, the genetic backgrounds for growth traits, including cell multiplication and cell differentiation or metabolic pathways such as biomineralization, can play a critical role during graft maturation and pearl formation [49, 50].

### Objectives

In this study, we aimed to investigate the molecular basis conditioning growth performance in the pearl oyster *P. margaritifera* by studying differential gene expression between fast- and slow-growing phenotypes of juveniles from the same bi-parental cohort. *Furthermore*, gene expression profiles were also compared with the genome polymorphism from coding regions using variant annotation and mutational effect prediction. Here, we expect that putative mutations associated with differentially regulated genes may also be related to the phenotypic differences observed among oysters. Overall, this study provides valuable new insight on the role played by the molecular component is P. *margaritifera* growth performance.

## Materials and methods

### Animals and tissue sampling

An F2 cohort was generated from a bi-parental oyster family and maintained at Regahiga Pearl farm (Mangareva atoll, Gambier archipelago, French Polynesia). This biological material was chosen to reduce the genetic background noise in order to clarify the response at the molecular level, e.g., the contribution of epistasis to inter-individual variation was alleviated. Larvae were settled in controlled hatchery tanks using mussel rope collectors. After 5.5 months of rearing, the largest (*n* = 10) and smallest (*n* = 10) individuals, representing the head and the tail of the size distribution were selected as “Fast-growing” (hereafter referred to as F) and “Slow-growing” (hereafter referred to as S) phenotypes (Table S1). These twenty selected oysters were then dissected and their whole fresh tissues individually collected and preserved in RNAlater^®^ at -80 °C until RNA extraction.

### RNA extraction, library preparation, and sequencing

Total RNA extraction was performed on the whole soft tissues of animals using TRIzol^®^ reagent (Invitrogen), following manufacturer’s recommendations. Total RNA integrity was assessed on a Bioanalyzer 2100 (Agilent Technologies, USA). Total RNA was dried in RNA-stable solution (Thermo Fisher Scientific) following manufacturer’s instructions and shipped at room temperature to McGill sequencing platform services (Montreal, Canada). RNA-seq libraries were generated using an Illumina TruSeq RNA Sample preparation kit according to manufacturer’s instructions (Illumina). RNA libraries were multiplexed (*n* = 10 individual libraries per sequencing lane) and sequenced on an Illumina HiSeq 4000 to produce 100-bp paired-end reads.

### Quality control and sequence data preprocessing

First, low-quality reads, short reads, and Illumina adapters were removed or trimmed using TrimGalore! v.0.6.4 9 ([51] https://github.com/FelixKrueger/TrimGalore*)* (q > 30, length > 50 bp). Sequence quality was assessed using FastQC v.11.9 [52]. Reads were aligned with STAR v.2.7.9a [53], using the draft genome available for *P. margaritifera* (draft genome available under the European Nucleotide Archive Accession PRJEB73564). The output BAM files were filtered for uniquely mapped reads, with mapping quality ≥ 30, then sorted and indexed using SAMtools v.1.9 [54]. Htseq-count v.0.9.1 [55] was used to count the reads that mapped to a single gene model annotated in the *P. margaritifera* GFF file. For downstream analyses, we carried out two filtering steps in order to reduce signal noise; (1) low coverage transcripts with less than five samples showing more than ten reads were discarded, (2) transcripts showing abnormally elevated proportion of reads (> 5%) compared with the total read count among a sample were excluded.

### Data analysis

#### Differential expression analysis and sample-based clustering

After the filtering steps, the raw counts of the remaining genes were normalized applying the medians of ratios method available in the R package DESeq2 v.1.26.0 [56]. To assess the overall covariation of gene expression among samples, we performed variance stabilizing transformation (VST) of gene counts in DESeq2 and principal component analysis (PCA) across the top 50 most variables genes. We then performed differential expression analysis to compare the F and S groups. Here, DESeq2 determined differentially expressed genes (DEGs) using a Wald test followed by Benjamini & Hochberg false discovery rate (FDR) correction. Genes were considered as DEG if the FDR (adjusted *P* value) was < 0.01 and the absolute log_2_ fold change was > 1.5.

#### Functional annotation and gene ontology enrichment

Gene ontology (GO) annotations for all genes identified in the reference genome were obtained by using BLAST V1.1 against the Swissprot/Uniprot database. In total, the background gene list used to perform our gene ontology enrichment analysis contained 13,674 annotated genes. Gene ontology enrichment analysis for DEGs was performed using a ranked-based gene ontology analysis implemented using the GO_MWU approach (GO analysis using adaptive clustering and Mann-Whitney U-test, as in [57]. Such GO term enrichment analysis is usually applied to provide further information about molecular functions (MF), biological processes (BP) and cellular components (CC). Herein, analysis was performed using the results of gene expression profiling in the form of signed negative log *P* values. Significance of functional GO terms was assessed from ten random permutations and we set a minimum of five genes for any individual GO term to be considered. Similar GO terms were also merged (gene-sharing pattern) by fixing a *clusterCutHeight* parameter at 0.5. Finally, only GO terms significantly enriched with an adjusted *P* value < 0.01 were considered as relevant.

#### Gene co-expression network analysis

We used weighted gene co-expression network analysis (WGCNA, R package v.1.7-3; [58] to identify modules of highly co-expressed genes. WGCNA was performed using the VST matrix of gene counts exported from DESeq2 as described above. Genes showing low variance (i.e., σ^2^ < 0.10) were also discarded to reduce noise and increase computing efficiency. As a result, the input matrix contained 16,900 genes for the signed WGCNA network building. Modules were identified using the *blockwiseModules* function implemented in the WGCNA R package [58]. Here, the soft thresholding power value (β) was set at 12, the value at which the network reaches scale-free topology. All function parameters were kept at default values except for *minModuleSize*, *mergeCutHeight* and *deepsplit*, which were set at 100, 0.30, and 1, respectively. Then, module eigengenes (ME, corresponding to the first principal component of a given module) were calculated using the *moduleEigengene* function of the WGCNA R package.

We tested putative associations between ME and growth phenotypes by calculating *Pearson* correlation coefficients. Modules showing a correlation value up to |0.6| and a *P* value < 0.01 were considered significantly correlated with the experimental variable. Hub genes were also screened in significant modules using the gene significance (GS, which is the correlation between individual genes and experimental variables) and module membership (MM, which is the correlation between individual genes and ME). In this way, if GS and MM show a high correlation value, this means that the genes represent the most important elements of a module and are highly significantly associated with experimental variable [59, 60]. GO enrichment analysis was also performed for the key modules significantly associated with the growth phenotypes using the same approach described above for DEGs.

#### Sequence polymorphism survey of DEGs

To study putative associations between sequence polymorphisms and gene expression, SNPs were called across all individuals and annotated for the potential functional effects of allelic variation. To do this, uniquely mapped reads for each of the individual BAM files were filtered for PCR duplicates using Picard Mark Duplicates (Picard Toolkit, 2019. https://broadinstitute.github.io/picard/*).* Then, variant calling was performed with BCFTools v.1.4.1 [61], using the default parameters. To retain high confidence SNPs, the raw SNP dataset was filtered with BCFtools using the command: --include “TYPE = ’snp’ & N_ALT = 1 & minDP ≥ 10 & maxDP ≤ 2000 & minQAL ≥ 100 & MapQ ≥ 30 & AN > 30 & AC ≥ 3” --SnpGap 10, which keeps all SNPs that pass the following criteria: (1) variant type is biallelic SNP, (2) keep SNP if all samples have between a minimum of 10 reads a maximum of 2000 reads, (3) a m Minimum Phred quality score of 100 and minimum Map Quality score of 30, (5) total number of called alleles more than 30 (i.e. max missing rate allowed ~ 20%), (6) minor allele count of three and (7) discard SNPs closer than 10 bp to an INDEL. Functional effect annotation of SNPs was then predicted with SnpEff v.5.0 [62]. The SnpEff database was built using the same unpublished genome assembly as that used for RNA-seq read mapping. Only SNPs that were included within pre-identified DEGs were retained and variants were flagged with warning annotations (e.g., from incomplete, incorrect or low accuracy predictions) were also discarded. Finally, we searched for candidate SNPs associated with growth phenotypes using a redundancy analysis (RDA). Candidate SNPs for significant association were identified based on a cut-off of ± 2.75 standard deviations (*P* value < 0.006) from the mean loading of the first RDA axis.

#### Alternative splicing analysis

Analyzing alternative splicing (AS) in the presence of SNP within a transcript can be critical as site mutation can affect splice site recognition, leading to altered splicing pattern for intance. Hence, splicing alterations can be associated with the production of aberrant protein isoforms which potentially affect cellular function. In contrast, mutational effect of a SNP can be mitigated or overlooked by AS events, where different splice patterns can compensate for or bypass mutational impact, maintaining normal protein function or expression levels. Here, we assess AS patterns using two complementary approaches, generalized linear models implemented in the R package DEXseq [63] and Multivariate Analysis of Transcript Splicing implemented in the program rMATS [64].

For DEXseq, low expressed exons were discared, keeping only thoses where at least ten reads were aligned to a minimum of ten samples. Normalization of exon read count was performed using DESeq2 algorithm as suggested by DEXseq manual recommendation. Differential exon usage was assessed based on an FDR threshold of 0.01. While DEXseq allow only detection of differential exon usage, rMATS identifies and quantifies the major types of alternative splicing patterns, including skipped exons, alternative 5’ and 3’ splice sites, mutually exclusive exons, and retained introns. Here rMATS was performed using STAR sorted-BAM files using a pre-filtering step to retain only reads that were mapped to the scaffold43000 (113.025 kbp). False discovery rate < 0.01 and p-value < 0.05 were fixed as level of significance.

## Results

### Biometry

Shell surface area was significantly different between F and S *P. margaritifera* juveniles (Wilcoxon *P* value < 0.001, Table S1). The average shell surface ranged from 107 to 188 mm^2^ (average 146 ± 25.7 mm^2^) and from 63 to 88 mm^2^ (average 76 ± 9.9 mm^2^) for the F and S groups, respectively.

### Sequencing, mapping, and filtering

Among the twenty samples for which RNA was sequenced, one F sample showed a very low yield of sequencing data and was removed before any pre-processing steps. Mean raw reads reached 30.07 +/- 7.39 M and 29.28 +/- 7.07 M were retained after filtering for quality (Table S1). All samples showed Q30 frequencies of bases higher than 96% and standard GC contents (40%), indicating good quality of the RNA sequencing reads. Overall, about 65.1–66.5% of reads were uniquely mapped to the *P. margaritifera* draft genome, with an average of 23% and 11% of multi-mapping and unmapped reads, respectively (Table S2). In total, from the 72,433 genes identified, 46.43% passed the post-quality filtering criteria, leading to a final set of 33,635 genes for downstream analyses.

### Differential expression analysis

**Fig. 1:**
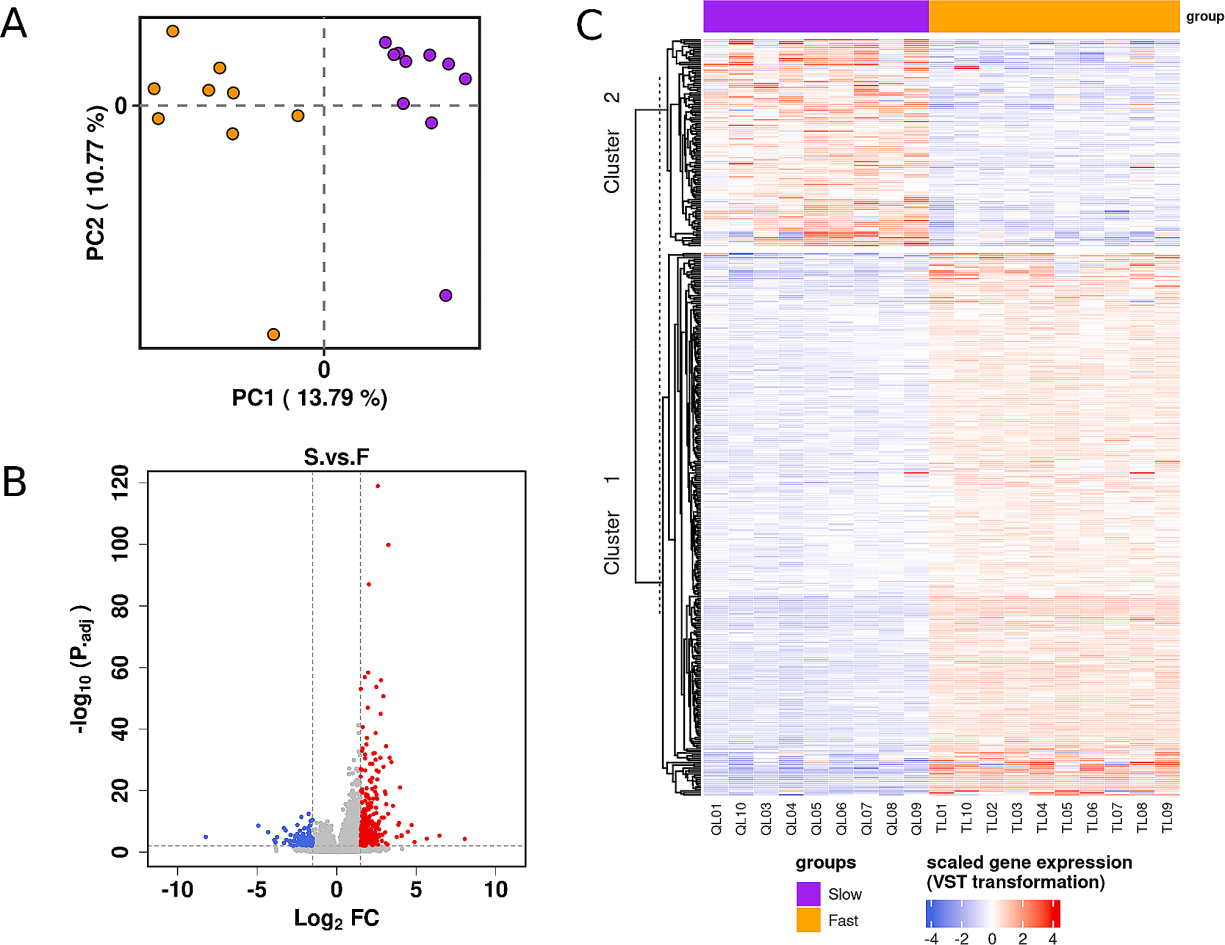
Analysis of gene expression profiles between *P. margaritifera* juveniles groups for Fast- and Slow-growing phenotypes. (**A**) Two-dimensional PCA plot of complete gene expression across all samples (*n* = 19). Each point represents an individual colored according to its phenotype (F: orange; S: purple). (**B**) Volcano plot of gene expression data depicting differentially expressed genes (DEGs) between the oyster groups. Red dots represent up-regulated DEGs for the F phenotype and blue dots represent up-regulated DEGs for the S phenotype. Grey doted lines represent the threshold limits of DEGs identification (|Log_2_FC| > 1.5 and FDR > 0.01). (**c**) Heatmap of the 394 DEGs identified by DESeq2 analysis. DEGs (rows) are grouped by cluster assignment based on a K-means algorithm. Color-coding of gene expression is based on read counts normalized by the variance stabilizing transformation (VST). Oysters are grouped in columns

Preliminary principal component analysis (PCA) of the top 500 most variable genes showed that the F and S oyster groups to be highly differentiated along the first PC axis, which explained 19.79% of the overall variance (Fig. 1A). Next, comparison between F and S revealed 394 differentially expressed genes (DEGs), with |logFC| > 1.5 and adjusted *P* value < 0.01 (Fig. 1B). Among these DEGs, 285 were up-regulated for F phenotype and 109 were up-regulated for S phenotype. Individuals’ gene expression profiles for the 394 DEGs are represented by a heatmap in Fig. 1C. Overall, the K-means clustering algorithm applied across gene expression profiles supported two clusters, in which up- and down-regulated genes are distinguished according to the DESeq analysis. The top10 DEGs (ranked according to the adjusted *P* value) for each phenotype group are given in Table 1. The complete results on the 394 DEGs have been made available in the Supplementary materials Table S3.

**Table 1:** List of the top 10 successfully annotated up-regulated DEGs in Fast- (F) and Slow- (S) growing *P. margaritifera* F2 juveniles. Gene IDs and descriptions were obtained from Blastn against Uniprot database. Log2FC: Log2-fold change

Transcript ID	Log_2_FC	log_10_(FDR)	Gene ID	Gene Description
**Top 10 up-regulated DEGs in F**				
scaffold8696size123454.3	3.409	Inf	P23098	Dynein beta chain, ciliary
scaffold2610size137834.3	2.587	118.983	Q6ZR08	Dynein axonemal heavy chain 12
scaffold2240size97364.3	3.252	99.852	Q8TE73	Dynein axonemal heavy chain 5
scaffold4322size93551.2	2.021	87.029	O15943	Neural-cadherin
scaffold8696size123454.2	1.980	58.353	P39057	Dynein beta chain, ciliary
scaffold9079size64000.2	1.768	56.878	Q8WXX0	Dynein axonemal heavy chain 7
scaffold4722size240932.3	2.788	55.890	Q8WXX0	Dynein axonemal heavy chain 7
scaffold2254size106444.9	2.496	53.684	Q80ZA4	Fibrocystin-L
scaffold2003size102676.2	1.546	53.052	Q9JJC8	Magnesium transporter NIPA2
scaffold3047size151625.8	2.936	50.654	Q9P2D7	Dynein axonemal heavy chain 1
**Top 10 up-regulated DEGs in S**				
scaffold54size490924.9	-1.518	-10.429	Q61483	Delta-like protein 1
scaffold5252size132102.1	-4.922	-8.578	Q9NQ29	Putative RNA-binding Luc7-like 1
scaffold494size163391.2	-2.095	-5.933	C8YR32	Lipoxygenase homology domain-containing protein 1
scaffold6618size92625.3	-2.481	-5.852	P27658	Collagen alpha-1(VIII) chain
scaffold896size230373.6	-2.895	-5.569	Q964E2	Actin
scaffold54size490924.6	-1.730	-5.562	Q61483	Delta-like protein 1
scaffold3907size124695.1	-2.342	-5.100	C8YR32	Lipoxygenase homology domain-containing protein 1
scaffold754size393405.3	-1.853	-4.649	Q61483	Delta-like protein 1
scaffold8185size41456.2	-1.812	-4.528	C8YR32	Lipoxygenase homology domain-containing protein 1
scaffold2007size114573.3	-1.931	-4.274	Q60847	Collagen alpha-1(XII) chain

### GO enrichment analysis of gene expression

The rank-based gene ontology analysis reported a total of 406 significantly enriched (FDR 0.01) terms in the comparison between the Fast- and Slow-growing phenotypes. This included 65, 257, and 84 terms belonging to Molecular Function (MF), Biological Process (BP), and Cellular Component (CC), respectively. We give the major GO terms that best represent “independent” groups of significant GO terms for gene expression differences between the Fast- and Slow-growing phenotypes in Table 2. Note that the complete results of our GO enrichment analysis are provided in the Supplementary materials. Broadly, we found that gene ontology for F oysters was mostly enriched with terms pertaining to growth and tissue homeostasis, including many pathways regulating cell proliferation, migration, differentiation, apoptosis, and motility (e.g., GO:0042058; GO:0032006; GO:0000902; GO:0004445). Additionally, several GO terms were associated with innate immune response (GO:1,900,424), mostly involving ubiquitin-like protein transferase activity. Contrastingly, gene ontology for S oysters was enriched for terms associated with DNA/RNA activity (GO:0003723; GO:0006351; GO:0065004), mitochondrial-related processes (GO:0006414; GO:0010257; GO:0098798), and structural constituent of ribosomes (GO:0003735; GO:0022613; GO:1,990,904).

**Table 2:** Gene ontology enrichment analysis. This table presents the GO terms that best represent “independent” groups of significant GO terms for gene expression differences between the fast- and slow-growing phenotypes. See supplementary material for complete results of the gene ontology enrichment analysis. Gene ratio indicates the number of significant genes (*P* value < 0.01) present in a category/total genes belonging to this category. FDR values correspond to an adjusted *P* value cutoff of 0.05 using the Benjamini–Hochberg method

GO terms	GO id	Gene ratio	FDR	regulation
**Ontology: Molecular Function**				
inositol phosphate phosphatase activity	GO:0004445	9/22	6.63e-04	Up
signaling receptor complex adaptor activity	GO:0030159	12/31	5.72e-04	Up
cytoskeletal motor activity	GO:0003774	66/141	3.00e-10	Up
phosphatidylinositol binding	GO:1,901,981	87/256	3.87e-11	Up
SH3 domain binding	GO:0017124	42/108	6.43e-04	Up
GTPase binding	GO:0031267	216/608	6.35e-16	Up
protein kinase activity	GO:0004672	162/463	8.32e-12	Up
ubiquitin-like protein transferase activity	GO:0004842	118/397	6.02e-06	Up
calcium ion binding	GO:0005509	107/337	7.94e-05	Up
cytoskeletal protein binding	GO:0008092	290/891	4.78e-06	Up
transcription regulator activity	GO:0140110	279/995	1.46e-04	Down
Catalytic activity, acting on a nucleic acid	GO:0140640	126/670	3.83e-05	Down
RNA binding	GO:0003723	200/966	2.33e-20	Down
peptide receptor activity	GO:0001653	46/110	1.47e-07	Down
oxidoreduction-driven active transmembrane transporter	GO:0003954	13/58	1.84e-05	Down
structural constituent of ribosome	GO:0003735	41/164	2.33e-20	Down
**Ontology: Biological Processes**				
astrocyte differentiation	GO:0048708	21/45	3.24e-05	Up
regulation of defense response to bacterium	GO:1,900,424	20/68	3.92e-04	Up
protein autophosphorylation	GO:0018105	112/318	6.01e-17	Up
regulation of phosphatidylinositol 3-kinase signaling	GO:0014066;	39/91	2.46e-04	Up
mucopolysaccharide metabolic process	GO:1,903,510	27/132	5.58e-06	Up
regulation of ERBB signaling pathway	GO:0042058	53/159	1.56e-06	Up
regulation of TOR signaling	GO:0032006	41/141	1.72e-04	Up
axo-dendritic transport	GO:0008088	60/146	1.97e-04	Up
regulation of dephosphorylation	GO:0010921	67/177	2.96e-04	Up
protein polyubiquitination	GO:0000209	89/289	1.56e-06	Up
endomembrane system organization	GO:0010256	137/557	2.21e-04	Up
cell morphogenesis	GO:0000902	450/1336	5.15e-07	Up
RNA biosynthetic process	GO:0006351	186/730	1.73e-05	Down
protein-DNA complex subunit organization	GO:0065004	53/238	9.03e-05	Down
RNA processing	GO:0006396	247/1127	3.96e-35	Down
ribonucleoprotein complex biogenesis	GO:0022613	145/591	6.79e-20	Down
neuropeptide signaling pathway	GO:0007218	46/110	1.37e-08	Down
mitochondrial gene expression	GO:0006414	41/189	5.33e-16	Down
mitochondrial respiratory chain complex assembly	GO:0010257	14/64	2.52e-06	Down
regulation of postsynaptic cytosolic calcium ion	GO:0099566	7/11	3.64e-04	Down
**Ontology: Cellular Components**				
endosome	GO:0005768	255/870	1.05e-15	Up
apical part of cell	GO:0045177	188/632	7.06e-09	Up
perinuclear region of cytoplasm	GO:0048471	235/807	1.38e-10	Up
cytoplasmic region	GO:0099568	239/693	7.62e-08	Up
chromosome	GO:0005694	218/980	6.66e-06	Down
ribonucleoprotein complex	GO:1,990,904	241/978	1.43e-25	Down
peptidase complex	GO:0000502	23/125	2.73e-06	Down
preribosome	GO:0030684	28/133	2.72e-08	Down
mitochondrial protein-containing complex	GO:0098798	51/248	3.04e-20	Down
ribosome	GO:0005840	54/227	5.65e-19	Down

### Gene expression modules strongly correlated with growth phenotype

**Fig. 2:**
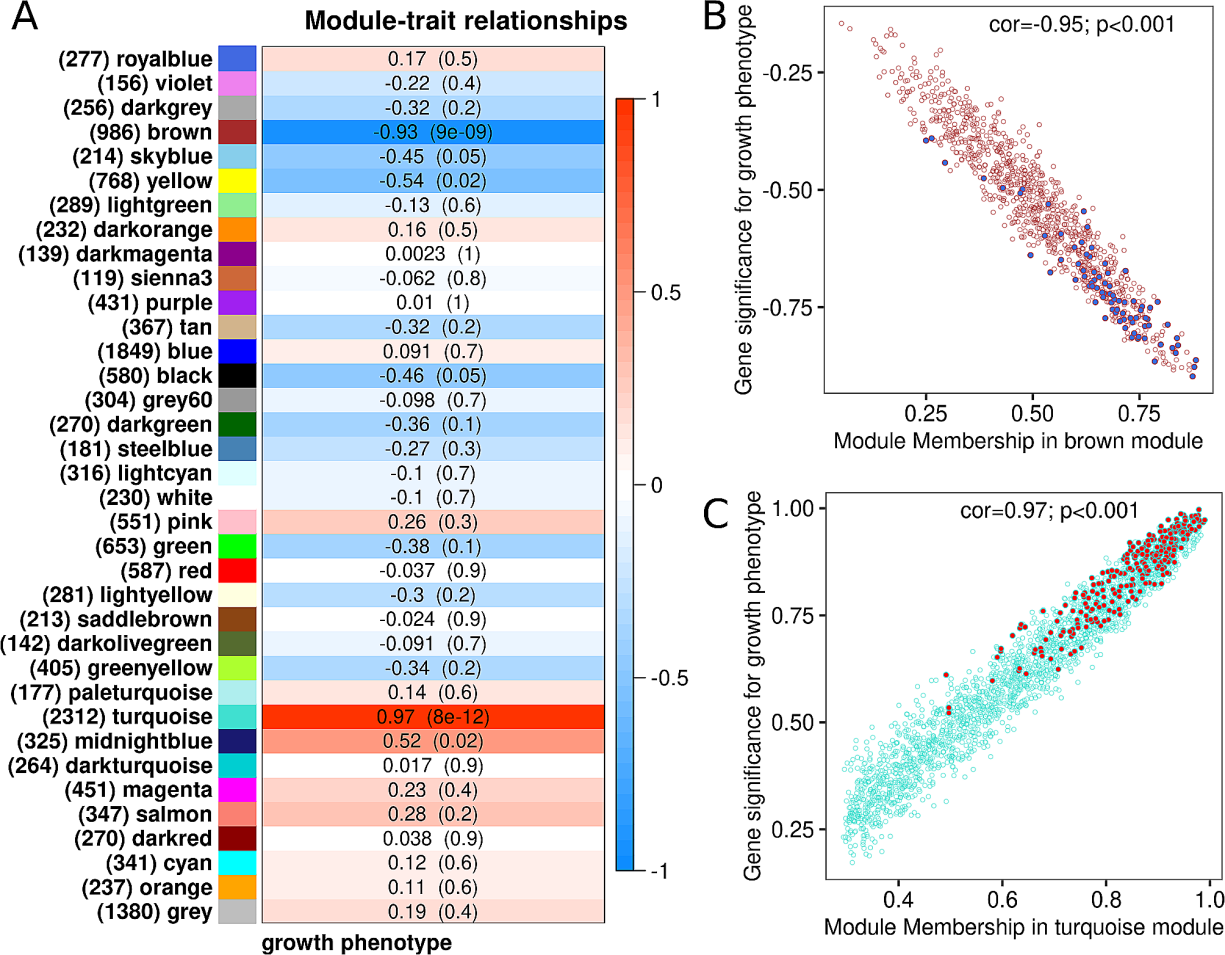
Correlation between gene modules and growth phenotypes in *P. margaritifera* juveniles. (**A**) WGCNA module-trait associations comparing module eigengene (gene count) to growth phenotype. Each cell contains the corresponding Pearson correlation value and its associated *P* value. The table is color-coded by correlation according to the legend. (**B**) Scatterplot of gene significance for growth phenotype vs. module membership in the brown module. (**C**) Scatterplot of gene significance for growth phenotype vs. module membership in the turquoise module. For **B** and **C**, filled circles represent DEGs previously identified by DESeq2 analysis where blue and red colors refer to up-regulated DEGs in S and F phenotypes respectively

**Fig. 3:**
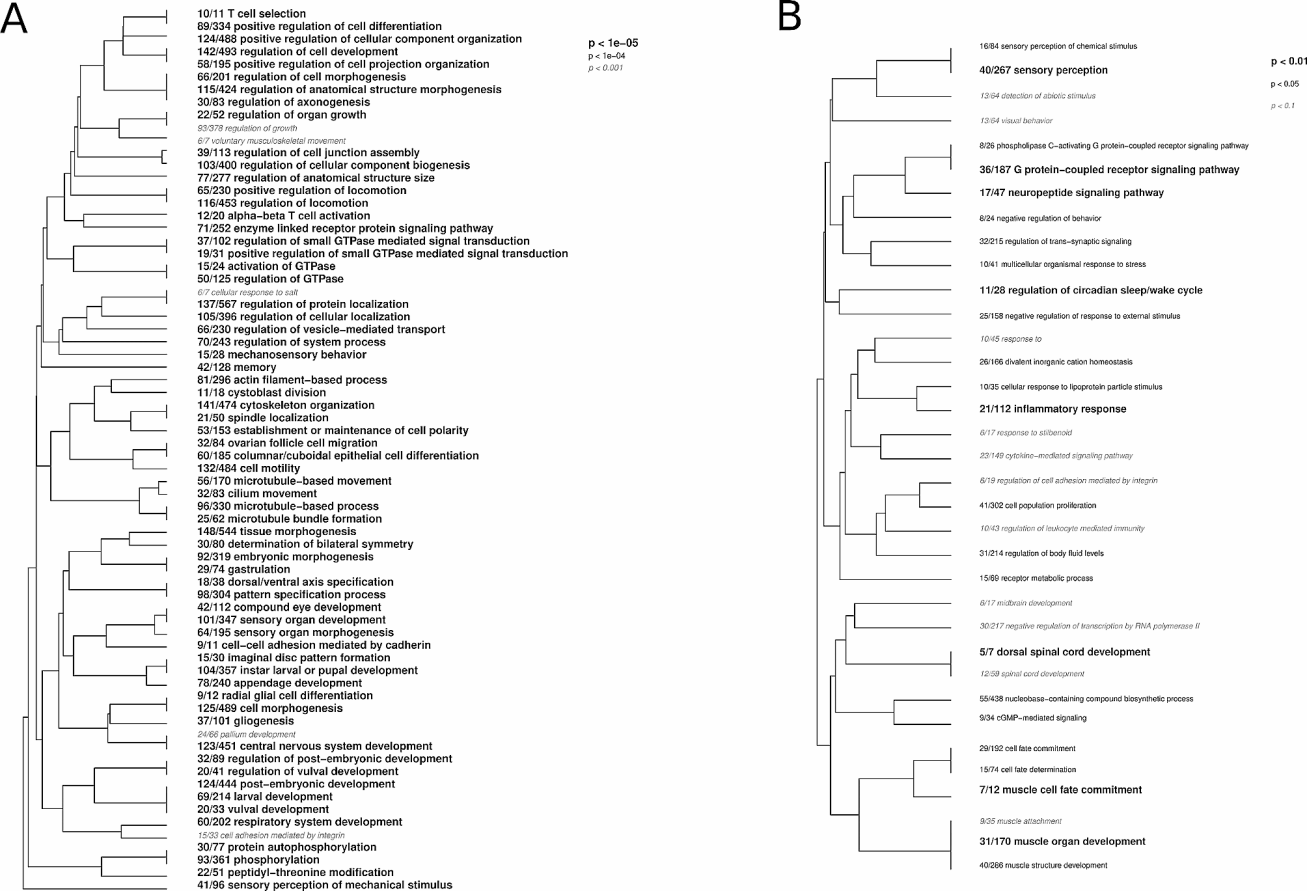
Gene Ontology (GO) enrichment analysis of WGCNA modules associated with *P. margaritifera* growth phenotype. Hierarchical clustering of Gene Ontology (GO) terms showing significant enrichment in the turquoise (**A**) and brown (**B**) modules. Only the biological process category is represented here. Level of significance is indicated with bold text

The co-expression network was constructed using 16,900 selected genes out of 33,635 (genes with low variability were discarded) and clusters of highly co-expressed genes (modules) were identified and assigned to color annotation modules, as shown in Fig. 2A. Overall, WGCNA identified 36 modules containing between 119 and 1,380 genes. Among these, two modules were strongly correlated with growth phenotype: the brown module (986 genes, r_pearson_ = -0.93, *P* value < 0.01) and the turquoise module (2,312 genes, r_pearson_ = 0.97, *P* value < 0.01). Furthermore, gene intra-modular connectivity of GS and MM for each selected module revealed that most of the genes showing high module membership had been identified as DEGs (Fig. 2B-C). We found that 362 (92%) DEGs belonged to one of the two significant modules, Brown and Turquoise, in which 88 down- and 276 up-regulated DEGs were distributed, respectively. Functional enrichment analysis of the Turquoise module highlighted strongly representative terms involving regulation of developmental condition and growth, from cell level to organ morphogenesis (Fig. 3A). Note that many enriched GO terms showed a high level of significance (*P* value < 1.e^-05^). In contrast, the brown module showed less functional enrichment than the turquoise module. Enriched GO terms were mainly associated with response to stimulus or stress, cell signaling pathways and immune response (Fig. 3B). The complete results on functional enrichment pertaining to the two significant WGCNA modules are provided in the Supplementary materials.

### Genome polymorphism associated with gene expression

Overall, our polymorphism survey of RNA-seq reads identified 990,536 filtered SNPs, in which 2,244 were distributed within the DEGs. Among these, 422 (18.8%) SNPs showed putative incorrect prediction from SNPeff analysis (i.e., warning annotation) and were excluded from downstream analyses. From the remaining 1,822 SNPs, SNPeff annotation reported 17 (1%), 698 (38.3%), 94 (5.1%), and 1013 (55.6%) SNPs that were assigned to high, moderate, modifier, and low impact categories, respectively.

**Fig. 4:**
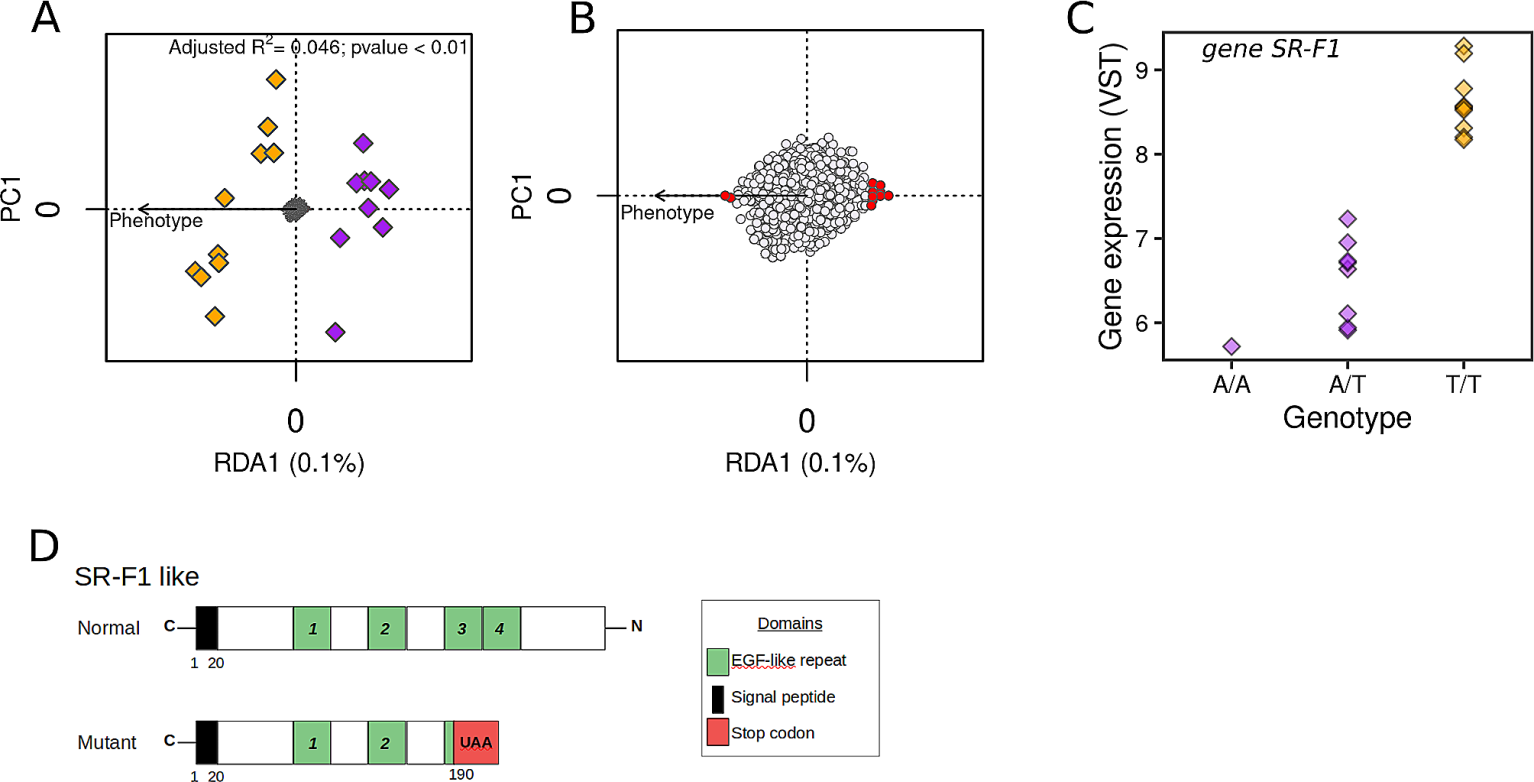
Analysis of gene polymorphism in *P. margaritifera*. (**A**) Redundancy analysis (RDA) performed with 1,824 SNPs called for 19 individuals using growth phenotype as the constraining variable on the first ordination axis. Grey points in the center of the plot represent SNPs, while colored diamonds represent individuals with orange and purple colors indicating Fast- (F) and Slow- (S) growing phenotypes respectively. (**B**) RDA biplot focusing on SNPs, where candidates for significant association (± 2.75 SD; *P* < 0.006) with growth phenotype are colored in red. (**C**) Genotype distribution vs. gene expression for the candidate SNP observed in the SR-F1 gene (scaffold4300size113025; base position 20,247) and associated with a high impact effect due to a stop codon gained mutation (see Table 3). Colored diamonds represent individuals, with orange and purple colors indicating Fast- (F) and Slow- (S) growing phenotypes, respectively. (**D**) Structural representation and motif composition of the SR-F1 like protein. Functional domains are based on Interpro protein predictive model database [65]. EGF: epidermal growth factor-like domain. Sequence base localization of domains are indicated below each scheme

Redundancy analysis identified 16 SNPs showing a significant association with growth phenotype (RDA, adjusted R^2^ = 0.046; ANOVA *P* value < 0.04; Fig. 4A-B). From these 16 candidate SNPs, eight were classified as synonymous (low impact category), seven were classified as missense variants (moderate impact category) and one was classified as a stop codon gained (high impact category; Table 3).

**Table 3:** Candidate SNPs within DEGs associated with Fast- (F) and Slow- (S) growing phenotypes of *P. Margaritifera* juveniles from RDA. A cut-off of ± 2.75 SD (*P* < 0.006) from the mean loading of the first axis (RDA1) was used to identify candidate SNPs from a two-tailed normal distribution where the mean was centered on 0

Scaffold	Position	Effect	Impact	RDA_loading	Log2FC	Padj	Gene_ID	Description	Up-Regulation
scaffold2377size95048	35,421	synonymous	Low	0.25	1.959	1.12E-47	DYH5_HUMAN	Dynein axonemal heavy chain 5	F
scaffold3047size151625	123,277	synonymous	Low	0.28	2.392	6.77E-33	DYH1_HUMAN	Dynein axonemal heavy chain 1	F
scaffold3047size151625	128,652	synonymous	Low	0.25	2.936	2.22E-51	DYH1_HUMAN	Dynein axonemal heavy chain 1	F
scaffold3333size191570	81,288	synonymous	Low	0.26	3.106	3.82E-35	XM_022477301.1	dynein cytoplasmic 1 heavy chain 1	F
scaffold3500size108302	87,376	synonymous	Low	0.28	-1.947	8.12E-04	NA		S
**scaffold4300size113025**	**20,247**	**stop_gained**	**High**	**-0.29**	**2.502**	**1.85E-19**	**SREC_MOUSE**	**Class F scavenger receptor SR-F1**	**F**
scaffold4300size113025	20,254	missense	Moderate	0.29	2.502	1.85E-19	SREC_MOUSE	Class F scavenger receptor SR-F1	F
scaffold4300size113025	20,475	missense	Moderate	0.29	2.502	1.85E-19	SREC_MOUSE	Class F scavenger receptor SR-F1	F
scaffold4300size113025	20,748	missense	Moderate	0.29	2.502	1.85E-19	SREC_MOUSE	Class F scavenger receptor SR-F1	F
scaffold4300size113025	20,520	missense	Moderate	0.29	0.502	1.85E-19	SREC_MOUSE	Class F scavenger receptor SR-F1	F
scaffold6914size72805	36,739	missense	Moderate	0.25	3.984	9.35E-22	CAS4_EPHMU	Short-chain collagen C4	F
scaffold6914size72805	36,752	synonymous	Low	0.32	3.984	9.35E-22	CAS4_EPHMU	Short-chain collagen C4	F
scaffold6914size72805	36,793	missense	Moderate	-0.32	3.984	9.35E-22	CAS4_EPHMU	Short-chain collagen C4	F
scaffold6914size72805	38,277	missense	Moderate	0.25	3.984	9.35E-22	CAS4_EPHMU	Short-chain collagen C4	F
scaffold7129size47494	32,807	synonymous	Low	0.28	-2.041	1.99E-08	NA		S
scaffold8696size123454	24,578	splice_region/synonymous	Low	0.25	3.409	0.00E + 00	DYHC_TRIGR	Dynein beta chain	F

Candidate SNPs were associated with three classes of annotated genes involving Dynein heavy chain, short-chain collagen C4 proteins, and scavenger receptor class F1 (SR-F1) activity. Focusing on the highest impact mutation (i.e., stop codon gained within the gene SR-F1; scaffold4300size113025, position 20,247), we found a strong association between genotypes and gene expression (ANOVA F value = 104.8; *P* value < 0.01; note that we excluded the individuals that were uniquely called for the reference genotype). Interestingly, we observed that all F oysters exhibited an alternative homozygous genotype for the stop codon gained mutation while all S oysters (except one) were heterozygous (Fig. 4C). Those homozygous for the stop-codon mutation (i.e., F oysters) showed higher SR-F1 expression compared with heterozygous (i.e., S oysters). Moreover, note that the unique S sample genotyped for the homozygous reference allele (i.e., absence of stop codon gained) also demonstrated the lowest gene expression for this transcript. Functional domain analysis from the InterPro database predicted that the position of this stop gain mutation might induce the loss of two out of four epidermal growth factor-like domains (EGF-like) observed along the N-terminal part of the protein (Fig. 4D).

### Analyses of alternative splicing

Following differential expression analysis at gene level, we studied putative splicing variations between fast- and slow-growing oyster groups. Among the 31,285 exons analyzed with DEXseq, 316 AS events showed significant differential exon usage (FDR < 0.01). However, no significant AS event was detected in exon profiles associated with the stop-codon mutation identified above (i.e. stop codon gained within the gene SR-F1; scaffold4300size113025, position 20,247). Analysis of AS conducted with rMATS revealed a potential exon skipping event in a gene unrelated to the stop-codon mutation investigated in this study. Moreover, after FDR correction, this putative AS event did not retain statistical significance.

## Discussion

Growth is an important economic trait in aquaculture and has been a priority focus in developing selective breeding programs. In the present study, we conducted a transcriptomic analysis to improve our understanding of the gene expression and genomic bases underlying inter-individual growth differences in *P. margaritifera*. Our results show substantial differences in terms of up- or down-regulated genes between Fast- and Slow-growing phenotypes that indicate the putative molecular mechanisms associated with this complex polygenic trait. Comparison of whole transcriptome profiles between the Fast- and Slow-growing oysters of our study allowed us to identify 394 DEGs, many of which were involved in growth-like biological processes. Additionally, a genome polymorphism survey identified a strong putative causal mutation within a gene (SR-F1) that plays a key role in apoptosis and tissue homeostasis.

### Molecular signal for growth

In the Fast-growing oysters, we mainly observed up-regulation for growth metabolism. Functional analysis showed enrichment of genes related to signal transduction, cell proliferation, cell division, cell motility. Notably, our findings highlighted increased cytoskeleton activity, for which genes pertaining to molecular motors such as microtubules (e.g., dynein, fibrocystin, kinesin, myosin, cadherin) were mostly up-regulated in F oysters. Microtubules are critical for a large number of cellular processes and mainly involved in maintaining cell structure and cytoskeleton as well as the cell movement process through by micro- or intermediate filaments [66]. Moreover, microtubules also form the major element of cilia and flagella, which are highly developed in bivalves and cover the external part of multiple tissues (i.e., mantle, gills, digestive gland). Overall, these results observed in F oysters are consistent with previous studies conducted on marine mollusks such as *C. gigas* [39], *Haliotis rufescens* [67] and *Mytilus galloprovincialis* [68]. However, this latter observation is expected because a faster growth rate is also usually accompanied by a general increase in the metabolism, particularly cellular division and tissue morphogenesis/ development. Our results also reported functional enrichment for regulation of cell death/ removal, otherwise known as apoptosis (i.e., regulation of ErbB and phosphatidylinositol signaling). For instance, ErbB family proteins are mostly cell surface receptors forming a pleiotropic signaling system [69]. One of the best described functions of ErbB receptor family comprises interaction with epidermal growth factors (EGF) ligands regulating aspects of cell proliferation, cell movement, and cell survival [69]. Interestingly, differences of gene expression between large and small pearl oysters involving the apoptosis pathway have already been observed in the closely related pearl oyster species *Pinctada fucata* [30]. Here, the authors reported a DEG annotated for an inhibitor of apoptosis 2 protein and one gene annotated for C1q domain-containing (C1qDC) proteins. Other studies conducted in bivalves have also reported up-regulation of genes related to apoptosis and association with fast-growth phenotypes [34, 70]. Taken together, our results show that the Fast-growing oysters seem to exhibit up-regulation patterns associated with tissue development and homeostatic growth. However, several studies underline a downside of such a phenotype, whereby increased growth rate may create a more favorable environment for faster development of pathogens, and thus a greater sensitivity to diseases [18].

### Trade-off for resource allocation between growth and immune function

Slow-growing oysters showed functional enrichment for up-regulated genes involved in transcription factors (i.e., synthesis of ribosomal elements), as well as mitochondrial metabolism. Interestingly, such metabolic contrast between Fast- and Slow-growing phenotypes has already been observed in previous studies on mollusk species [35, 39, 71–73]. In particular, Meyer and Manahan, 2010 reported that slow-growing oysters showed less homogeneous gene expression of ribosomal-related genes than fast-growing ones. Here, the authors argued for the “balance hypothesis”, which underpins a relationship between the stoichiometry of the most abundant cell metabolism proteins and whole-organism growth or fitness [74]. Although ribosomes are essential for growth and general development in eukaryotes, their synthesis is a costly process that requires close-coordination between ribosomal RNA (rRNA) synthesis and production of ribosomal proteins [74]. A nucleolar stress (also called “ribosomal stress”), which can be induced by cell stress or genome mutations, may affect rRNA synthesis and, in turn, crowd the protein synthesis in the cell. Consequently, deregulation of the ribosomal metabolic equilibrium is detrimental to the cell cycle as it can disrupt protein production machinery, enhance inflammatory signaling, and affect major cell processes such as cell homeostasis, cell proliferation, and growth [75]. Furthermore, ribosomes are also involved in biological infection, where intracellular pathogens such as viruses are able to induce heterogeneous rRNA production [27]. Thus, ribosomal stress through biological infection is also a suggested cell signaling pathwaythat can induce innate immune responses by increasing inflammatory protein synthesis [76]. Considering this latter hypothesis, we observed that the top gene expression profiles in S oysters revealed overexpression of genes known to be involved in immune and cell stress response. For instance, we found that three highly regulated transcripts were annotated for lipoxygenase-like proteins in S oysters. Lipoxygenases have been commonly observed to mediate inflammation events or pathogen exposure [77–79]. In addition, three other top up-regulated genes in the S oysters were annotated for delta-like protein 1 (DLK1). The family of delta-like proteins are molecular ligands identified to interact with cellular receptors of Notch signaling, an essential growth regulatory pathway of cell proliferation, differentiation, and apoptosis critical in metazoan development [80]. In marine invertebrates, Notch signaling pathways can also play a critical role in the regulation of innate immune responses to stress [81]. Taken together, our results suggest that the Slow-growing oysters show increased metabolic expression for immune activity compared with the Fast-growing ones. According to the theory of resource allocation, maintaining immune performance and growth, as well as other life-history traits is energetically demanding and has physiological costs [82, 83]. Such concurrent needs lead to trade-offs for resource allocation, which can be observed at the individual level though plastic modulations of physiological processes, and at the evolutionary level by genetic variation among individuals in a population. However, enhanced expression of genes associated with mitochondrial metabolism in S oysters may suggest a common pattern of cell response to stressful stimuli, which usually requires increase of energy production to enable cell adaptation [84]. In consequence, it may be that observed slow growth associated with enhanced immune function could result from stressful conditions during the rearing period. For instance, we cannot avoid putative heterogeneity for food availability within our rearing system. While food availability is known to influence the physiological condition of organisms, a number of studies support the general hypothesis that restricted food supply can promote expression of immune functions at the expense of growth [18, 85].

### Polymorphism affecting growth

In the present study, we found one SNP located within a gene annotated with Scavenger Receptor class F member 1 (SR-F1), which is predicted to present a high mutational effect (i.e., premature stop codon gained). Moreover, SR-F1 was expressed at significantly higher level in F oysters, which were all homozygous for the premature stop-codon mutation. The receptor SR-F1 is a highly evolutionary conserved protein, which contains extracellular domains showing significant sequence homology in the animal kingdom [86]. SR-F1 is well known to plays a key role in the clearance of apoptotic cells, making this receptor a critical control of tissue homeostasis [87]. Indeed, programmed cell death (apoptosis) and apoptotic corpse clearance by phagocytosis are described as a compensatory response during organism growth [88].

Mutation for a premature stop codon in the gene body can result in dramatic changes in the resulting protein (e.g., abnormally shortened, folding disturbance). These changes can lead to different consequences such as (*i*) the loss of functionality, (*ii*) neofunctionalization, (*iii*) disturbance with a compensatory response, or (*iv*) change in protein properties (e.g., stability, binding affinity, activity, localization, protein-protein interactions) [89]). Functionality loss is frequently illustrated in human research where premature stop codons are known to result in a large number of human diseases [90]. In our study, we hypothesize that the observed stop-codon mutation does not result in a total loss of SR-F1 protein function. Indeed, serious disturbance in the SR-F1 pathway would lead to more drastic deleterious consequences [87].

In contrast, up-regulation of SR-F1 in F oysters (homozygous mutants) suggests two putative hypotheses. First, mutation for premature stop codons would imply a negative impact, disturbing the capacity of SR-F1 to accomplish its role and leading to a compensatory response by over-expression of this gene. This phenomenon has already been reported in human, where an N-terminal truncating mutation caused by a premature stop codon on the erythropoietin receptor (EPOR) leads to hypo-responsiveness of erythropoietin (EPO), but normal hemoglobin concentration [91]. Overexpression of SR-F1 has already been investigated in human cell lines [92]. An important result was that up-regulation of SR-F1 led to a decrease of phagocytosis efficiency of apoptotic cells, which may interfere with tissue homeostasis (equilibrium between cell proliferation and apoptosis). However, this observation should be considered with caution, as homology or direct comparison between human cell lines and oysters remains tenuous.

A second hypothesis implies a positive impact of the stop-codon mutation. This less common instance has been reported in several cases of human EPOR mutation, promoting athletic performance [93], or in plants, where it is associated with improved growth traits [94]. For instance, two premature stop-codon mutations have been described in the TCP gene family in wheat, which play an important role in plant development and growth [94]. Interestingly, the authors found that stop-codon mutations led to increased spike and grain lengths, which may be helpful in genetic selection for wheat yield improvement.

Here, our results from functional domain analysis of SR-F1 suggest that the observed stop-codon mutation would eliminate two EGF-like repeats from the N-terminal region of the SR-F1 receptor. Similar mutations were investigated by [92] in human cell lines. Interestingly, they demonstrated that various N-terminal truncations (i.e., removing two to five EGF-like repeats) increased binding properties of the SR-F1 receptor, particularly its affinity to bind the C1qDC ligand, an important bridging protein for recognition of apoptotic cells. Even if the *P. margaritifera* SR-F1 gene sequence varies for the number of EGF-like repeats compared with its homologous sequence in other species, we can speculate that the binding property of the SR-F1 stop-codon mutant would be conserved and may participate in apoptosis clearance during tissue homeostasis and growth. Functional modifications of SR-F1 properties (e.g., increased binding) in the oyster model have not been demonstrated, so further studies are required to clarify this issue. Furthermore, post-transcriptional mechanisms (i.e., alternative splicing) by which transcriptome and proteome plasticity can be modulated also participate in transcript-level modifications and protein-level alterations [95]. Our results did not identified significant signal of alternative splicing event within the coding region of the SR-F1 gene. However, our sequencing data, similarly to most RNA-seq data are restricted to short reads < 200 bp, while alternative splicing events often occur in larger windows [96]. Moreover, whereas short reads are often aligned with multiple genome locations, they also commonly span a small number of exons, which adds substantial bias for splice graph analyses. Combined with the small number of samples included in the present study, these limitations prevent us from properly investigating alternative splicing for such mutations. Further work which can implement complementary data including long-read sequencing as well as qRT-PCR validation could enhance our understanding of potential regulatory mechanisms governing transcript expression through alternative splicing [97].

### Limitations of the study

Although we have been able to gain insight into the role of gene expression in inter-individual growth differences within the same oyster cohort, various factors could have confounded our interpretations. First, RNA analyses were conducted using whole oyster soft tissues. While this experimental approach represents the most convenient way of sampling from such small juvenile oysters, subtle but significant signals of differential gene expression may be masked by the effect size of well-represented tissues. In other words, if localized structures (e.g., epithelial ridge) or specific organs particularly involved in the studied trait are small relative to the whole soft tissues, large differences in gene expression within these structures/ organs may be hidden by other transcriptomic patterns [98]. For instance, the mollusk mantle is anatomically split into different regions responsible for secreting various layers of the shell (i.e., periostracum, prisms, or nacre) and previous transcriptomic analyses have shown that different genes are expressed in separate, discrete, and sometimes very limited regions of the mantle outer epithelium [99]. Nevertheless, we think that, in the present study, we were able to capture the main patterns of gene expression differences related to the growth phenotypes considered. Indeed, growth is a global biological trait, which affects all tissues and organs of an individual. Another issue is the presently incomplete annotation of the reference genome, which remains a key limitation to our capacity to investigate the complex and polygenic components of growth in *P. margaritifera* more deeply. Nevertheless, our study shows that valuable detail as well as overall patterns can be highlighted from transcriptomic profiles between well-differentiated phenotypes.

The SRF1 polymorphism reported in this study was strongly associated with the growth phenotypes and may represent a major causal variant for this trait. While strong phenotypic variation can be associated with one or a few large-effect loci [100], growth, like most biological traits, is highly polygenic [14]. Furthermore, it should be noted that our study investigated only one breeding family, which represents a restricted genetic background. Thus, we consider that the observed association between SRF1 polymorphism and growth phenotype needs to be studied further to draw conclusions on its real impact. Hence, the generalization of our findings pertaining to the SRF1 mutation is still limited. Additional studies based on multiple breeding families are required to investigate how genetic diversity and polygenic architecture can influence phenotype expression of growth in *Pinctada margaritifera*.

## Conclusion

In this study, we investigated differential gene expression of growth-related phenotypes in an F2 full-sib family of pearl oyster spat. Comparison of transcriptomic profiles showed that the difference between Fast- and Slow-growing oysters might be related to the balance of regulation between stress response and growth control pathways. Furthermore, analysis of sequence polymorphism identified a SNP annotated for a stop-codon mutation that possibly has a function in pearl oyster growth. Expression analysis for the associated gene showed a significantly higher expression level in the Fast-growing group, in which all individuals exhibited a homozygous genotype for the stop codon. Overall, this study provides valuable genomic resources for understanding the molecular bases regulating growth in *P. margaritifera* and other marine bivalve species.

### Electronic supplementary material

Below is the link to the electronic supplementary material.

Supplementary Material 1

## Data Availability

The raw sequence data (individual FASTQ files) have been deposited in the European Nucleotide Archive (ENA) under the project accession PRJEB72853. The P. margaritifera draft genome used to align RNA-seq reads is available under ENA project accession PRJEB73564. To ensure reproducibility of analyses, source code for the complete bioinformatic process has been deposited in a dedicated GitLab Digital Repository for this study, permanently archived at: https://rmpf.gitlab-pages.ifremer.fr/pmargaritifera_growth_rnaseq/.
