# Supplementary material for: Comparative transcriptomics identifies genes underlying growth performance of the Pacific black-lipped pearl oyster Pinctada margaritifera
Source: BMC Genomics. 2024 Jul 24;25:717. doi: 10.1186/s12864-024-10636-0 (PMC11270918; doi:10.1186/s12864-024-10636-0)
Supplement: Supplementary file 1 — Supplementary Material 1 [file 12864_2024_10636_MOESM1_ESM.docx]

**Supplementary Material to “Comparative transcriptomics identifies genes underlying growth performance of the Pacific black-lipped**

**pearl oyster *Pinctada margaritifera*”**

Y. Dorant^1,2^, V. Quillien^1,3^, J. Le Luyer^1,3^, C. L. Ky^1,2^

^1^Ifremer, ILM, IRD, UPF, UMR 241 SECOPOL, Taravao, Tahiti, Polynésie française, France.

^2^Interactions Hôtes Pathogènes Environnements (IHPE), Université de Montpellier, CNRS, Ifremer, Université de Perpignan Via Domitia, Montpellier, France.

^3^Ifremer, Univ Brest, CNRS, IRD, UMR 6539, LEMAR, F-29280, Plouzane, France.

* Corresponding author: Y. Dorant

**Supplementary Tables**

**Table S1.** **Biometric measurements on hatchery produced *Pinctada margaritifera* of 5.5 months old.** Shell surface area was estimated using standard image analysis techniques (ImageJ 1.5t, National Institute of Health, Washington DC).

| Sample* | Shell surface (mm^2^) |
| --- | --- |
| BH_01 | 152.8 |
| BH_02 | 171.5 |
| BH_03 | 124.2 |
| BH_04 | 161.5 |
| BH_05 | 127.4 |
| BH_06 | 158.7 |
| BH_07 | 118.5 |
| BH_08 | 149.1 |
| BH_09 | 188.2 |
| BH_10 | 107.4 |
| BT_01 | 63.3 |
| BT_02 | 70.2 |
| BT_03 | 87.1 |
| BT_04 | 81.1 |
| BT_05 | 88.8 |
| BT_06 | 70.9 |
| BT_07 | 83.2 |
| BT_08 | 63.0 |
| BT_09 | 67.2 |
| BT_10 | 83.6 |

*BH = Batch head (i.e., Fast-growing); BT = batch tail (i.e., Slow-growing)

**Table S2. Summary statistics of RNA-seq raw data and STAR mapping results**

|  | **Mean (n = 19)** | **SD (n = 19)** | **Min (n = 19)** | **Max (n = 19)** |
| --- | --- | --- | --- | --- |
| Number of raw reads | 30,074,983.79 | 7,394,813.83 | 21,269,546 | 57,225,391 |
| Number of quality reads | 29,281,539.16 | 7,074,570.53 | 20,766,471 | 55,055,321 |
| Average input read length | 195.80 | 0.71 | 193 | 196 |
| **UNIQUE READS** |  |  |  |  |
| Uniquely mapped reads number | 19,293,011.74 | 4,605,504.30 | 13,605,949 | 36,028,178 |
| % Uniquely mapped reads | 65.91 | 0.43 | 65.15 | 66.49 |
| Average mapped length | 193.62 | 0.61 | 191.3 | 194.0 |
| Number of splices: Total | 10,064,783.53 | 2,433,070.97 | 7,078,336 | 18,763,744 |
| Number of splices: Annotated (sjdb) | 10,016,121.05 | 2,416,938.69 | 7,044,565 | 18,651,422 |
| % Mismatch rate per base | 1.15 | 0.03 | 106.0 | 1.20 |
| Deletion rate per base | 0.10 | 0 | 0.1 | 0.1 |
| Deletion average length | 1.98 | 0.02 | 2.0 | 2.0 |
| Insertion rate per base | 0.05 | 0 | 0.04 | 0.05 |
| Insertion average length | 2.07 | 0.06 | 1.91 | 2.15 |
| **MULTI-MAPPING READS** |  |  |  |  |
| Number of reads mapped to multiple loci | 6,649,638.11 | 1,707,324.67 | 4,784,752 | 12,876,834 |
| % of reads mapped to multiple loci | 22.66 | 0.56 | 21.6 | 23.8 |
| Number of reads mapped to too many loci | 166,856.47 | 47,050.75 | 118,710 | 345,802 |
| % of reads mapped to too many loci | 0.57 | 0.03 | 0.5 | 0.63 |
| **UNMAPPED READS** |  |  |  |  |
| Number of reads unmapped: too many mismatches | 24.11 | 13.74 | 9 | 74 |
| % of reads unmapped: too many mismatches | <0.5 | <0.5 | <0.5 | <0.5 |
| Number of reads unmapped: too short | 3,063,544.11 | 698,551.88 | 2,172,989 | 5,583,993 |
| % of reads unmapped: too short | 10.49 | 0.30 | 10.12 | 11.29 |
| Number of reads unmapped: other | 108,464.63 | 29,904.03 | 74,571 | 220,440 |
| % of reads unmapped: other | 0.37 | 0.02 | 0.34 | 0.4 |
| **CHIMERIC READS** |  |  |  |  |
| Number of chimeric reads | 0 | 0 | 0 | 0 |
| % of chimeric reads | 0 | 0 | 0 | 0 |

**Table S3. Description of the 394 DEGs identified.**

Gene IDs and descriptions were obtained from Blastn against Uniprot database. Log2FC: Log2-fold change. Positive and negative values of Log2FC means up-regulation in Fast (F) and Slow (S)-growing oysters respectively.

| **Transcript ID** | **Log_2_FC** | **log_10_(FDR)** | **Gene ID** | **Gene Description** |
| --- | --- | --- | --- | --- |
| evm.TU.scaffold8696size123454.3 | 3.41 | 0 | P23098 | Dynein beta chain, ciliary |
| evm.TU.scaffold2610size137834.3 | 2.59 | 1.04E-119 | Q6ZR08 | Dynein axonemal heavy chain 12 |
| evm.TU.scaffold2240size97364.3 | 3.25 | 1.41E-100 | Q8TE73 | Dynein axonemal heavy chain 5 |
| evm.TU.scaffold4322size93551.2 | 2.02 | 9.35E-88 | O15943 | Neural-cadherin |
| evm.TU.scaffold8696size123454.2 | 1.98 | 4.43E-59 | P39057 | Dynein beta chain, ciliary |
| evm.TU.scaffold9079size64000.2 | 1.77 | 1.32E-57 | Q8WXX0 | Dynein axonemal heavy chain 7 |
| evm.TU.scaffold4722size240932.3 | 2.79 | 1.29E-56 | Q8WXX0 | Dynein axonemal heavy chain 7 |
| evm.TU.scaffold2254size106444.9 | 2.5 | 2.07E-54 | Q80ZA4 | Fibrocystin-L |
| evm.TU.scaffold2003size102676.2 | 1.52 | 8.86E-54 | Q9JJC8 | Magnesium transporter NIPA2 |
| evm.TU.scaffold3047size151625.8 | 2.94 | 2.22E-51 | Q9P2D7 | Dynein axonemal heavy chain 1 |
| evm.TU.scaffold2377size95048.4 | 1.96 | 1.12E-47 | Q8TE73 | Dynein axonemal heavy chain 5 |
| evm.TU.scaffold1size567472.12 | 2.77 | 1.17E-45 | E1BUG7 | Protein SPT2 homolog |
| evm.TU.scaffold6723size50050.1 | 1.64 | 2.30E-41 | NA | NA |
| evm.TU.scaffold2012size135527.3 | 2.46 | 2.04E-39 | A2ARV4 | Low-density lipoprotein receptor-related protein 2 |
| evm.TU.scaffold10448size30907.1 | 1.9 | 8.01E-38 | Q6ZTR5 | Cilia- and flagella-associated protein 47 |
| evm.TU.scaffold1808size385059.5 | 1.88 | 1.02E-35 | Q80ZA4 | Fibrocystin-L |
| evm.TU.scaffold3333size191570.5 | 3.11 | 3.82E-35 | NA | NA |
| evm.TU.scaffold7082size88875.2 | 1.62 | 1.83E-34 | Q93008 | Probable ubiquitin carboxyl-terminal hydrolase FAF-X |
| evm.TU.scaffold923size253024.5 | 1.59 | 1.27E-33 | Q5VT06 | Centrosome-associated protein 350 |
| evm.TU.scaffold3047size151625.7 | 2.39 | 6.77E-33 | Q9P2D7 | Dynein axonemal heavy chain 1 |
| evm.TU.scaffold2061size149152.5 | 2.3 | 8.59E-33 | NA | NA |
| evm.TU.scaffold2628size90693.1 | 1.78 | 1.90E-32 | Q4G0P3 | Hydrocephalus-inducing protein homolog |
| evm.TU.scaffold7734size44023.6 | 3.34 | 2.23E-31 | NA | NA |
| evm.TU.scaffold3333size191570.4 | 2.75 | 2.23E-31 | Q9JHU4 | Cytoplasmic dynein 1 heavy chain 1 |
| evm.TU.scaffold4731size141069.1 | 1.79 | 3.26E-31 | Q9TU34 | nositol 1,4,5-trisphosphate receptor type 1 |
| evm.TU.scaffold19size471443.12 | 2.44 | 9.86E-31 | Q6NYU2 | Probable helicase with zinc finger domain |
| evm.TU.scaffold503size162414.2 | 3.44 | 5.55E-30 | NA | NA |
| evm.TU.scaffold627size186339.4 | 2.24 | 2.04E-29 | NA | NA |
| evm.TU.scaffold748size144005.3 | 2.1 | 2.14E-29 | NA | NA |
| evm.TU.scaffold9871size125518.7 | 2.95 | 1.91E-28 | NA | NA |
| evm.TU.scaffold432size238846.13 | 1.52 | 9.61E-28 | P98161 | Polycystin-1 |
| evm.TU.scaffold1195size218953.14 | 1.57 | 1.77E-27 | NA | NA |
| evm.TU.scaffold1size567472.11 | 2.51 | 2.69E-27 | NA | NA |
| evm.TU.scaffold7998size108004.4 | 1.79 | 2.69E-27 | B3EWY9 | Mucin-like protein |
| evm.TU.scaffold2772size131853.6 | 2.64 | 6.90E-27 | Q9Y5G3 | Protocadherin gamma-B1 |
| evm.TU.scaffold2216size247672.21 | 1.51 | 2.68E-25 | P10079 | Fibropellin-1 |
| evm.TU.scaffold1039size131048.1 | 2.24 | 7.83E-25 | P10040 | Protein crumbs |
| evm.TU.scaffold1094size184517.5 | 2.55 | 2.39E-24 | P25054 | Adenomatous polyposis coli protein |
| evm.TU.scaffold4722size240932.4 | 2.08 | 5.33E-24 | NA | NA |
| evm.TU.scaffold9079size64000.1 | 2.16 | 1.61E-23 | Q8WXX0 | Dynein axonemal heavy chain 7 |
| evm.TU.scaffold19size471443.11 | 2.43 | 7.94E-22 | Q6NYU2 | Probable helicase with zinc finger domain |
| evm.TU.scaffold6914size72805.2 | 3.98 | 9.35E-22 | P18503 | Short-chain collagen C4 |
| evm.TU.scaffold6785size49613.1 | 1.89 | 1.78E-21 | NA | NA |
| evm.TU.scaffold4242size117557.12 | 2.23 | 2.69E-21 | P78509 | Reelin |
| evm.TU.scaffold2596size91064.2 | 1.61 | 4.40E-21 | Q9TXQ1 | Poly [ADP-ribose] polymerase tankyrase |
| evm.TU.scaffold2383size182244.7 | 1.53 | 1.36E-20 | NA | NA |
| evm.TU.scaffold34size332373.12 | 3.04 | 2.00E-20 | Q8R508 | Protocadherin Fat 3 |
| evm.TU.scaffold2216size247672.23 | 2.42 | 2.25E-20 | Q4LDE5 | Sushi, von Willebrand factor type A, EGF and pentraxin domain-containing protein 1 |
| evm.TU.scaffold568size156750.3 | 1.62 | 4.64E-20 | P70227 | Inositol 1,4,5-trisphosphate receptor type 3 |
| evm.TU.scaffold11056size28501.1 | 3.07 | 1.27E-19 | NA | NA |
| evm.TU.scaffold9014size52480.3 | 1.85 | 1.51E-19 | Q3B8D5 | Katanin p60 ATPase-containing subunit A-like 2 |
| evm.TU.scaffold4300size113025.3 | 2.5 | 1.85E-19 | Q5ND28 | Scavenger receptor class F member 1 |
| evm.TU.scaffold5766size129656.4 | 1.91 | 2.44E-19 | Q99PW8 | Kinesin-like protein KIF17 |
| evm.TU.scaffold207size334503.7 | 1.94 | 2.68E-19 | NA | NA |
| evm.TU.scaffold1254size464210.6 | 1.85 | 8.27E-19 | P70206 | Plexin-A1 |
| evm.TU.scaffold1435size315887.5 | 1.98 | 1.55E-18 | O15943 | Neural-cadherin |
| evm.TU.scaffold13721size19393.1 | 2.48 | 3.17E-18 | P52633 | Signal transducer and transcription activator 6 |
| evm.TU.scaffold9232size107439.3 | 2.29 | 4.12E-18 | NA | NA |
| evm.TU.scaffold7952size76572.2 | 2.54 | 4.55E-18 | P0C6F1 | Dynein axonemal heavy chain 2 |
| evm.TU.scaffold1456size237848.8 | 2.06 | 4.55E-18 | NA | NA |
| evm.TU.scaffold13111size21319.1 | 1.79 | 2.03E-17 | B3EWZ5 | MAM and LDL-receptor class A domain-containing protein 1 |
| evm.TU.scaffold1470size203364.5 | 1.59 | 6.03E-17 | Q5T4S7 | E3 ubiquitin-protein ligase UBR4 |
| evm.TU.scaffold748size144005.4 | 2.19 | 9.36E-17 | NA | NA |
| evm.TU.scaffold1276size403367.19 | 1.59 | 9.81E-17 | Q14517 | Protocadherin Fat 1 |
| evm.TU.scaffold142size220728.6 | 2 | 1.51E-16 | Q8IVL1 | Neuron navigator 2 |
| evm.TU.scaffold5327size114497.5 | 1.94 | 2.09E-16 | Q13127 | RE1-silencing transcription factor |
| evm.TU.scaffold1430size117431.4 | 2.01 | 2.15E-16 | P78363 | Retinal-specific phospholipid-transporting ATPase |
| evm.TU.scaffold34size332373.13 | 3.11 | 2.53E-16 | NA | NA |
| evm.TU.scaffold4809size145212.3 | 2 | 4.27E-16 | Q8BW94 | Dynein axonemal heavy chain 3 |
| evm.TU.scaffold2061size149152.4 | 2.07 | 6.98E-16 | NA | NA |
| evm.TU.scaffold34size332373.11 | 3.54 | 8.92E-16 | Q8R508 | Protocadherin Fat 3 |
| evm.TU.scaffold3333size191570.6 | 2.19 | 2.30E-15 | P37276 | Dynein heavy chain, cytoplasmic |
| evm.TU.scaffold4080size159219.5 | 2.48 | 3.56E-15 | Q24498 | Ryanodine receptor |
| evm.TU.scaffold1414size117744.3 | 1.94 | 4.30E-15 | P52633 | Signal transducer and transcription activator 6 |
| evm.TU.scaffold1size567472.10 | 1.7 | 1.22E-14 | NA |  |
| evm.TU.scaffold3418size108535.1 | 2.01 | 1.38E-14 | Q5THJ4 | Intermembrane lipid transfer protein VPS13 |
| evm.TU.scaffold506size177798.4 | 1.7 | 1.96E-14 | NA | NA |
| evm.TU.scaffold5634size57725.1 | 1.69 | 2.59E-14 | Q9NZJ4 | Sacsin |
| evm.TU.scaffold3013size182610.5 | 1.84 | 2.77E-14 | NA | NA |
| evm.TU.scaffold7879size43198.1 | 1.74 | 5.94E-14 | D3YXS5 | Kinesin-like protein KIF28 |
| evm.TU.scaffold2301size281225.13 | 1.75 | 1.07E-13 | NA | NA |
| evm.TU.scaffold1448size288317.2 | 1.62 | 1.07E-13 | P34611 | B-box type zinc finger protein ncl-1 |
| evm.TU.scaffold6281size66852.1 | 1.62 | 1.25E-13 | Q8NFP9 | Neurobeachin |
| evm.TU.scaffold469size165284.6 | 2.5 | 1.86E-13 | NA | NA |
| evm.TU.scaffold240size211441.12 | 3.23 | 3.35E-13 | Q80X19 | Collagen alpha-1(XIV) chain |
| evm.TU.scaffold8288size40987.1 | -1.77 | 3.94E-13 | NA | NA |
| evm.TU.scaffold1636size111866.5 | 2.32 | 4.50E-13 | Q23551 | Twitchin |
| evm.TU.scaffold5930size122547.4 | 1.78 | 4.86E-13 | P90666 | Thioredoxin domain-containing protein 3 homolog |
| evm.TU.scaffold12873size22085.1 | 1.7 | 6.54E-13 | O94916 | Nuclear factor of activated T-cells 5 |
| evm.TU.scaffold8818size98203.1 | 1.69 | 9.39E-13 | Q9WV30 | Nuclear factor of activated T-cells 5 |
| evm.TU.scaffold8871size38104.1 | 1.77 | 1.33E-12 | NA | NA |
| evm.TU.scaffold2470size93471.5 | 1.79 | 1.66E-12 | P97526 | Neurofibromin |
| evm.TU.scaffold7754size43867.1 | 2.09 | 1.83E-12 | P55023 | Tyrosinase |
| evm.TU.scaffold1042size256941.4 | 1.58 | 1.94E-12 | NA | NA |
| evm.TU.scaffold3013size182610.6 | 1.76 | 3.67E-12 | NA | NA |
| evm.TU.scaffold2477size168662.5 | 1.65 | 3.77E-12 | NA | NA |
| evm.TU.scaffold4743size116923.6 | -2.22 | 3.83E-12 | NA | NA |
| evm.TU.scaffold9232size107439.1 | 1.78 | 4.51E-12 | Q9TU53 | Cubilin |
| evm.TU.scaffold12960size21824.1 | 2.83 | 5.72E-12 | Q18DN4 | Halomucin |
| evm.TU.scaffold1715size163509.10 | 2.97 | 6.67E-12 | Q9NZJ4 | Sacsin |
| evm.TU.scaffold444size167998.9 | 1.87 | 6.87E-12 | Q9C0G6 | Dynein axonemal heavy chain 6 |
| evm.TU.scaffold1705size160013.3 | 1.93 | 7.13E-12 | NA | NA |
| evm.TU.scaffold6492size72947.3 | 2.02 | 8.82E-12 | NA | NA |
| evm.TU.scaffold4198size112046.5 | 1.94 | 1.78E-11 | P33450 | Cadherin-related tumor suppressor |
| evm.TU.scaffold9762size65719.1 | 1.84 | 1.92E-11 | NA | NA |
| evm.TU.scaffold695size399694.6 | 1.67 | 3.18E-11 | NA | NA |
| evm.TU.scaffold54size490924.9 | -1.52 | 3.72E-11 | Q61483 | Delta-like protein 1 |
| evm.TU.scaffold9044size71881.4 | 1.64 | 6.30E-11 | Q3KR37 | Protein Aster-B |
| evm.TU.scaffold204size245622.7 | 1.96 | 6.76E-11 | Q9NRA2 | Sialin |
| evm.TU.scaffold466size489227.21 | 1.99 | 7.11E-11 | D3YXG0 | Hemicentin-1 |
| evm.TU.scaffold76size343677.22 | -1.63 | 7.74E-11 | NA | NA |
| evm.TU.scaffold1705size160013.4 | 2.67 | 9.13E-11 | Q9EQW7 | Kinesin-like protein KIF13A |
| evm.TU.scaffold5327size114497.6 | 2 | 9.81E-11 | P52746 | Zinc finger protein 142 |
| evm.TU.scaffold888size137471.3 | 2.2 | 1.01E-10 | NA | NA |
| evm.TU.scaffold5327size114497.7 | 1.83 | 1.29E-10 | NA | NA |
| evm.TU.scaffold9652size34378.1 | 1.57 | 1.57E-10 | Q14185 | Dedicator of cytokinesis protein 1 |
| evm.TU.scaffold1039size131048.2 | 1.78 | 1.91E-10 | P10079 | Fibropellin-1 |
| evm.TU.scaffold3085size133383.3 | 2.03 | 2.27E-10 | A2AWL7 | MAX gene-associated protein |
| evm.TU.scaffold695size399694.7 | 1.72 | 2.40E-10 | NA | NA |
| evm.TU.scaffold1425size146356.7 | 2.64 | 2.56E-10 | NA | NA |
| evm.TU.scaffold14152size18171.1 | 1.78 | 2.97E-10 | Q12923 | Tyrosine-protein phosphatase non-receptor type 13 |
| evm.TU.scaffold3158size118848.1 | 1.64 | 2.97E-10 | NA | NA |
| evm.TU.scaffold1768size150764.5 | 1.78 | 3.35E-10 | Q8CAI1 | Coiled-coil domain-containing protein 142 |
| evm.TU.scaffold7343size46253.2 | 3.92 | 3.53E-10 | NA | NA |
| evm.TU.scaffold874size205353.9 | 1.93 | 4.77E-10 | A0A0R4IBK5 | E3 ubiquitin-protein ligase rnf213-alpha |
| evm.TU.scaffold459size166490.3 | 2.35 | 4.98E-10 | NA |  |
| evm.TU.scaffold6684size114491.4 | 2.06 | 5.60E-10 | Q70CQ2 | Ubiquitin carboxyl-terminal hydrolase 34 |
| evm.TU.scaffold2772size131853.5 | 1.82 | 6.66E-10 | Q24292 | Protein dachsous |
| evm.TU.scaffold4918size90178.2 | 2.6 | 6.79E-10 | NA | NA |
| evm.TU.scaffold4198size112046.7 | 2.58 | 1.27E-09 | Q6V0I7 | Protocadherin Fat 4 |
| evm.TU.scaffold3626size158813.6 | 2.45 | 1.35E-09 | Q9GV77 | Extracellular matrix protein 3 |
| evm.TU.scaffold2960size121078.3 | 1.81 | 1.35E-09 | Q8R151 | NFX1-type zinc finger-containing protein 1 |
| evm.TU.scaffold7734size44023.7 | 3.88 | 1.39E-09 | NA | NA |
| evm.TU.scaffold4630size66424.2 | 1.75 | 1.47E-09 | Q6ZPY5 | Zinc finger protein 507 |
| evm.TU.scaffold6297size52881.4 | -2.47 | 1.59E-09 | NA |  |
| evm.TU.scaffold11498size41501.2 | 4.72 | 1.67E-09 | P40935 | Phenylethanolamine N-methyltransferase |
| evm.TU.scaffold6634size50621.3 | 1.75 | 1.78E-09 | NA | NA |
| evm.TU.scaffold2925size86034.2 | -1.67 | 2.16E-09 | NA | NA |
| evm.TU.scaffold8327size76228.1 | 1.55 | 2.54E-09 | NA | NA |
| evm.TU.scaffold5252size132102.1 | -4.92 | 2.64E-09 | Q9NQ29 | Putative RNA-binding protein Luc7-like 1 |
| evm.TU.scaffold2216size247672.17 | 1.98 | 2.89E-09 | NA | NA |
| evm.TU.scaffold13594size32124.2 | 2.27 | 3.31E-09 | Q9Y4D7 | Plexin-D1 |
| evm.TU.scaffold7734size44023.4 | 1.71 | 3.43E-09 | NA | NA |
| evm.TU.scaffold7033size72301.1 | 1.9 | 4.83E-09 | A2A891 | Calmodulin-binding transcription activator 1 |
| evm.TU.scaffold15731size13625.1 | 2.52 | 6.69E-09 | P0C6B8 | Sushi, von Willebrand factor type A, EGF and pentraxin domain-containing protein 1 |
| evm.TU.scaffold9086size42335.1 | 1.62 | 7.15E-09 | Q60989 | E3 ubiquitin-protein ligase XIAP |
| evm.TU.scaffold3421size79425.2 | 2.44 | 9.10E-09 | A0A0R4IBK5 | E3 ubiquitin-protein ligase rnf213-alpha |
| evm.TU.scaffold1964size188345.8 | -3.31 | 1.58E-08 | NA | NA |
| evm.TU.scaffold1225size153422.2 | -1.8 | 1.59E-08 | NA | NA |
| evm.TU.scaffold1553size230917.8 | 1.75 | 1.63E-08 | C8YR32 | Lipoxygenase homology domain-containing protein 1 |
| evm.TU.scaffold3903size146711.3 | -2.03 | 1.79E-08 | NA | NA |
| evm.TU.scaffold3size498981.17 | 1.53 | 1.89E-08 | Q5T1R4 | Transcription factor HIVEP3 |
| evm.TU.scaffold4084size113835.5 | 1.88 | 1.99E-08 | Q149M9 | NACHT domain- and WD repeat-containing protein 1 |
| evm.TU.scaffold7129size47494.4 | -2.04 | 1.99E-08 | NA | NA |
| evm.TU.scaffold880size137800.6 | 2.43 | 2.11E-08 | Q8NCA9 | Zinc finger protein 784 |
| evm.TU.scaffold1332size208501.2 | 1.54 | 3.43E-08 | E7FAM5 | E3 ubiquitin-protein ligase TRIM71 |
| evm.TU.scaffold204size245622.4 | 1.79 | 3.60E-08 | NA |  |
| evm.TU.scaffold500size256079.6 | 2.14 | 3.69E-08 | Q80TP3 | E3 ubiquitin-protein ligase UBR5 |
| evm.TU.scaffold4320size97644.2 | 1.53 | 3.91E-08 | D3Z7P3 | Glutaminase kidney isoform, mitochondrial |
| evm.TU.scaffold3213size82055.1 | 2.67 | 4.31E-08 | Q9PTY5 | Ran-binding protein 9 |
| evm.TU.scaffold746size316904.8 | 1.63 | 4.33E-08 | Q24498 | Ryanodine receptor |
| evm.TU.scaffold4198size112046.6 | 2.16 | 5.51E-08 | NA | NA |
| evm.TU.scaffold6884size48915.2 | 1.81 | 5.76E-08 | Q17R14 | Unconventional myosin-Id |
| evm.TU.scaffold2061size149152.3 | 1.53 | 5.81E-08 | NA |  |
| evm.TU.scaffold3387size79966.3 | 1.94 | 7.18E-08 | Q9MYM7 | Beta-1,3-galactosyltransferase 1 |
| evm.TU.scaffold5347size59947.4 | -1.74 | 7.72E-08 | NA |  |
| evm.TU.scaffold5868size56085.1 | 1.89 | 8.46E-08 | Q96KG7 | Multiple epidermal growth factor-like domains protein 10 |
| evm.TU.scaffold300size518482.25 | -3.21 | 8.69E-08 | NA | NA |
| evm.TU.scaffold15108size35241.1 | 1.82 | 1.03E-07 | NA | NA |
| evm.TU.scaffold7129size47494.3 | -2.32 | 1.11E-07 | NA | NA |
| evm.TU.scaffold11998size25021.1 | 1.62 | 1.13E-07 | Q9VT28 | Protein furry |
| evm.TU.scaffold11769size25833.1 | 1.97 | 1.15E-07 | Q99PW8 | Kinesin-like protein KIF17 |
| evm.TU.scaffold1070size154277.6 | 1.55 | 1.16E-07 | NA | NA |
| evm.TU.scaffold9453size35376.1 | 4.48 | 2.68E-07 | NA | NA |
| evm.TU.scaffold7727size44038.1 | -1.71 | 3.08E-07 | NA | NA |
| evm.TU.scaffold219size308910.8 | 1.79 | 3.17E-07 | NA | NA |
| evm.TU.scaffold300size518482.22 | -4.31 | 3.19E-07 | NA | NA |
| evm.TU.scaffold2992size85134.9 | 2.42 | 3.67E-07 | NA | NA |
| evm.TU.scaffold2261size156397.1 | 3.05 | 3.75E-07 | Q28983 | Zonadhesin |
| evm.TU.scaffold767size165377.10 | 2.2 | 3.81E-07 | Q811F1 | Zinc finger and BTB domain-containing protein 41 |
| evm.TU.scaffold880size137800.7 | 1.65 | 5.53E-07 | NA | NA |
| evm.TU.scaffold923size253024.6 | 1.98 | 6.24E-07 | Q5VT06 | Centrosome-associated protein 350 |
| evm.TU.scaffold3903size146711.2 | -1.78 | 6.28E-07 | NA | NA |
| evm.TU.scaffold12293size37448.1 | 1.73 | 6.89E-07 | NA | NA |
| evm.TU.scaffold2012size135527.1 | 2.18 | 9.66E-07 | NA | NA |
| evm.TU.scaffold512size279355.15 | 1.79 | 1.07E-06 | NA | NA |
| evm.TU.scaffold494size163391.2 | -2.1 | 1.17E-06 | C8YR32 | Lipoxygenase homology domain-containing protein 1 |
| evm.TU.scaffold6618size92625.3 | -2.48 | 1.41E-06 | P27658 | Collagen alpha-1(VIII) chain |
| evm.TU.scaffold3677size172094.6 | 2.22 | 1.58E-06 | NA | NA |
| evm.TU.scaffold45size373465.6 | 2.09 | 1.63E-06 | Q8VI56 | ow-density lipoprotein receptor-related protein 4 |
| evm.TU.scaffold512size279355.14 | 1.56 | 1.64E-06 | NA | NA |
| evm.TU.scaffold13997size18648.1 | 1.52 | 1.64E-06 | Q61555 | Fibrillin-2 |
| evm.TU.scaffold1151size340449.13 | 1.72 | 1.65E-06 | NA | NA |
| evm.TU.scaffold589size246915.12 | -1.75 | 1.71E-06 | NA | NA |
| evm.TU.scaffold1457size116792.2 | 2.05 | 1.76E-06 | Q8TDJ6 | DmX-like protein 2 |
| evm.TU.scaffold7912size56359.1 | 2.64 | 1.97E-06 | NA | NA |
| evm.TU.scaffold2655size358103.5 | 1.67 | 1.99E-06 | NA | NA |
| evm.TU.scaffold7734size44023.5 | 2.21 | 2.10E-06 | NA | NA |
| evm.TU.scaffold11584size52177.1 | 1.62 | 2.15E-06 | Q9DBB9 | Carboxypeptidase N subunit 2 |
| evm.TU.scaffold13660size19581.1 | 2.88 | 2.30E-06 | O95714 | E3 ubiquitin-protein ligase HERC2 |
| evm.TU.scaffold896size230373.6 | -2.9 | 2.69E-06 | Q964E2 | Actin, cytoplasmic |
| evm.TU.scaffold54size490924.6 | -1.73 | 2.74E-06 | Q61483 | Delta-like protein 1 |
| evm.TU.scaffold1414size117744.2 | 4.08 | 3.19E-06 | NA | NA |
| evm.TU.scaffold649size247893.9 | 1.51 | 3.23E-06 | NA | NA |
| evm.TU.scaffold159size303642.19 | 1.57 | 3.34E-06 | O75165 | DnaJ homolog subfamily C member 13 |
| evm.TU.scaffold1225size153422.7 | -1.74 | 4.08E-06 | NA | NA |
| evm.TU.scaffold3065size104404.3 | 2.48 | 4.46E-06 | NA | NA |
| evm.TU.scaffold14485size29345.1 | 6.46 | 4.46E-06 | Q24292 | Protein dachsous |
| evm.TU.scaffold998size180313.6 | 1.62 | 4.46E-06 | P49641 | Alpha-mannosidase 2x |
| evm.TU.scaffold1257size210641.5 | 1.63 | 4.71E-06 | Q9ERC5 | Otoferlin |
| evm.TU.scaffold1368size118871.6 | -2.6 | 4.88E-06 | NA | NA |
| evm.TU.scaffold6785size49613.2 | 1.58 | 4.97E-06 | P10079 | Fibropellin-1 |
| evm.TU.scaffold7247size46856.1 | 2.02 | 5.16E-06 | Q04833 | Low-density lipoprotein receptor-related protein |
| evm.TU.scaffold3250size127454.3 | 2.32 | 5.34E-06 | M9NDE3 | Protein bark beetle |
| evm.TU.scaffold1691size178697.5 | -1.69 | 5.38E-06 | NA | NA |
| evm.TU.scaffold3090size177860.6 | 1.51 | 5.86E-06 | Q5BKL9 | 45 kDa calcium-binding protein |
| evm.TU.scaffold3847size142878.6 | 4.08 | 5.95E-06 | NA | NA |
| evm.TU.scaffold3907size124695.1 | -2.34 | 7.94E-06 | C8YR32 | Lipoxygenase homology domain-containing protein 1 |
| evm.TU.scaffold152size248445.5 | 1.63 | 8.61E-06 | NA | NA |
| evm.TU.scaffold1277size177878.1 | -2.09 | 8.61E-06 | NA | NA |
| evm.TU.scaffold6343size86564.4 | 1.78 | 8.83E-06 | NA | NA |
| evm.TU.scaffold4083size180852.4 | 2.66 | 9.41E-06 | Q5H8C1 | FRAS1-related extracellular matrix protein 1 |
| evm.TU.scaffold2395size94680.4 | 1.94 | 9.41E-06 | G3MWR8 | F-actin]-monooxygenase MICAL3 |
| evm.TU.scaffold923size253024.7 | 1.66 | 9.79E-06 | NA | NA |
| evm.TU.scaffold1715size163509.9 | 2.03 | 1.02E-05 | Q9JLC8 | Sacsin |
| evm.TU.scaffold3315size80884.1 | 1.67 | 1.04E-05 | Q6H236 | Paternally-expressed gene 3 protein |
| evm.TU.scaffold2623size90776.2 | 2.56 | 1.04E-05 | NA | NA |
| evm.TU.scaffold670size250180.2 | -2.61 | 1.06E-05 | NA | NA |
| evm.TU.scaffold1170size125571.7 | 1.54 | 1.18E-05 | O00750 | hosphatidylinositol 4-phosphate 3-kinase C2 domain-containing subunit beta |
| evm.TU.scaffold723size192823.4 | 3.77 | 1.21E-05 | Q9P225 | Dynein axonemal heavy chain 2 |
| evm.TU.scaffold8629size110240.8 | 2.09 | 1.22E-05 | NA | NA |
| evm.TU.scaffold51size417995.10 | -8.23 | 1.24E-05 | NA | NA |
| evm.TU.scaffold300size518482.19 | -3.75 | 1.35E-05 | NA | NA |
| evm.TU.scaffold392size205118.1 | 1.99 | 1.58E-05 | Q498G2 | Centrosomal protein of 152 kDa |
| evm.TU.scaffold9968size32938.1 | -2.01 | 1.75E-05 | NA | NA |
| evm.TU.scaffold5141size122288.3 | -1.81 | 1.92E-05 | NA | NA |
| evm.TU.scaffold16551size11298.1 | 2.06 | 1.96E-05 | NA | NA |
| evm.TU.scaffold754size393405.3 | -1.85 | 2.24E-05 | Q61483 | Delta-like protein 1 |
| evm.TU.scaffold2133size250432.8 | 2.23 | 2.27E-05 | NA | NA |
| evm.TU.scaffold159size303642.18 | 1.87 | 2.38E-05 | O75165 | DnaJ homolog subfamily C member 13 |
| evm.TU.scaffold617size213831.9 | -2.06 | 2.45E-05 | NA | NA |
| evm.TU.scaffold240size211441.10 | 2.14 | 2.59E-05 | NA | NA |
| evm.TU.scaffold681size258651.12 | 1.54 | 2.61E-05 | NA | NA |
| evm.TU.scaffold5332size286306.21 | 2.37 | 2.80E-05 | NA | NA |
| evm.TU.scaffold1030size131372.1 | -1.72 | 2.82E-05 | NA | NA |
| evm.TU.scaffold853size172994.2 | 1.99 | 2.96E-05 | NA | NA |
| evm.TU.scaffold80size398458.6 | -1.75 | 2.96E-05 | NA | NA |
| evm.TU.scaffold8185size41456.2 | -1.81 | 2.96E-05 | C8YR32 | Lipoxygenase homology domain-containing protein 1 |
| evm.TU.scaffold3304size188632.6 | 1.94 | 3.10E-05 | NA | NA |
| evm.TU.scaffold3173size138203.1 | -1.71 | 3.12E-05 | NA | NA |
| evm.TU.scaffold16076size12687.1 | -1.52 | 3.32E-05 | NA | NA |
| evm.TU.scaffold6364size72041.2 | 1.73 | 3.34E-05 | Q967F4 | Cadherin-related hmr-1 |
| evm.TU.scaffold1026size247016.11 | 1.84 | 3.61E-05 | NA | NA |
| evm.TU.scaffold7047size62808.1 | 1.78 | 3.61E-05 | Q8CIQ7 | Dedicator of cytokinesis protein 3 |
| evm.TU.scaffold8888size38017.2 | -1.74 | 3.69E-05 | NA | NA |
| evm.TU.scaffold441size322675.5 | -2.38 | 3.70E-05 | NA | NA |
| evm.TU.scaffold11806size25663.1 | 2.11 | 3.77E-05 | NA | NA |
| evm.TU.scaffold2871size126566.10 | -1.57 | 4.22E-05 | NA | NA |
| evm.TU.scaffold7640size99468.2 | -1.5 | 4.32E-05 | NA | NA |
| evm.TU.scaffold9803size93631.2 | 5.67 | 4.35E-05 | A1ADJ6 | Polysialic acid O-acetyltransferase |
| evm.TU.scaffold1982size248760.5 | 1.55 | 5.01E-05 | Q9GL21 | Uveal autoantigen with coiled-coil domains and ankyrin repeats |
| evm.TU.scaffold2007size114573.2 | -1.88 | 5.03E-05 | NA | NA |
| evm.TU.scaffold657size202439.1 | 8.06 | 5.12E-05 | NA | NA |
| evm.TU.scaffold2007size114573.3 | -1.93 | 5.31E-05 | Q60847 | Collagen alpha-1(XII) chain |
| evm.TU.scaffold7129size47494.2 | -1.91 | 5.63E-05 | NA | NA |
| evm.TU.scaffold1276size403367.14 | 1.84 | 5.70E-05 | NA | NA |
| evm.TU.scaffold240size211441.9 | 1.98 | 6.02E-05 | NA | NA |
| evm.TU.scaffold6912size48766.1 | -1.74 | 6.18E-05 | NA | NA |
| evm.TU.scaffold3560size77635.1 | 2.01 | 6.73E-05 | P51589 | Cytochrome P450 2J2 |
| evm.TU.scaffold2133size250432.5 | -2.24 | 7.69E-05 | NA | NA |
| evm.TU.scaffold854size411799.9 | 1.79 | 7.91E-05 | NA | NA |
| evm.TU.scaffold7057size47893.2 | 2.48 | 8.13E-05 | NA | NA |
| evm.TU.scaffold5182size103520.3 | -1.86 | 8.33E-05 | NA | NA |
| evm.TU.scaffold3626size158813.5 | 2.31 | 8.60E-05 | NA | NA |
| evm.TU.scaffold293size338516.18 | 1.61 | 0.000104 | Q6INN8 | Deoxynucleoside triphosphate triphosphohydrolase SAMHD1 |
| evm.TU.scaffold6937size98117.3 | -1.56 | 0.000111 | NA | NA |
| evm.TU.scaffold3399size91464.1 | -3.16 | 0.000111 | NA | NA |
| evm.TU.scaffold1384size118527.1 | -3.92 | 0.00012 | NA | NA |
| evm.TU.scaffold6725size127633.6 | 2.5 | 0.000127 | NA | NA |
| evm.TU.scaffold1538size192633.6 | 1.93 | 0.000129 | NA | NA |
| evm.TU.scaffold5226size119279.1 | -2.12 | 0.000129 | NA | NA |
| evm.TU.scaffold2133size250432.6 | 1.98 | 0.000132 | NA | NA |
| evm.TU.scaffold3718size75908.3 | 1.81 | 0.000135 | Q80TP3 | E3 ubiquitin-protein ligase UBR5 |
| evm.TU.scaffold10083size32523.5 | 1.53 | 0.000163 | Q5SZK8 | FRAS1-related extracellular matrix protein 2 |
| evm.TU.scaffold2465size165730.2 | -2.66 | 0.000172 | NA |  |
| evm.TU.scaffold1320size225772.10 | 1.87 | 0.000189 | Q98ST7 | DBH-like monooxygenase protein 1 |
| evm.TU.scaffold3996size82558.5 | -1.56 | 0.000218 | P80457 | Xanthine dehydrogenase/oxidas |
| evm.TU.scaffold2308size186115.12 | 2.54 | 0.000218 | Q7TXL7 | Phenolphthiocerol/phthiocerol polyketide synthase subunit C |
| evm.TU.scaffold7413size45853.1 | -1.68 | 0.000218 | NA | NA |
| evm.TU.scaffold4324size150199.9 | 2.85 | 0.00023 | NA | NA |
| evm.TU.scaffold296size185649.11 | -1.75 | 0.00024 | NA | NA |
| evm.TU.scaffold4084size113835.4 | 1.58 | 0.000243 | NA | NA |
| evm.TU.scaffold321size182500.9 | -2.94 | 0.000257 | NA | NA |
| evm.TU.scaffold89size239972.7 | 1.87 | 0.000259 | Q8BW94 | Dynein axonemal heavy chain 3 |
| evm.TU.scaffold17592size7974.1 | -2.69 | 0.00027 | NA | NA |
| evm.TU.scaffold11509size26777.2 | -2.97 | 0.000272 | P02740 | Serum amyloid A protein |
| evm.TU.scaffold7279size46692.1 | 2.16 | 0.000272 | NA | NA |
| evm.TU.scaffold4793size129496.2 | 1.82 | 0.000309 | O01346 | Beta-1,4-mannosyltransferase egh |
| evm.TU.scaffold466size489227.20 | 1.75 | 0.000332 | D3YXG0 | Hemicentin-1 |
| evm.TU.scaffold4778size178960.1 | 2.19 | 0.000361 | Q15772 | Striated muscle preferentially expressed protein kinase |
| evm.TU.scaffold6486size51574.2 | 1.81 | 0.000369 | Q9ULK2 | Ataxin-7-like protein 1 |
| evm.TU.scaffold3562size90992.16 | 1.62 | 0.000373 | NA | NA |
| evm.TU.scaffold3653size253576.4 | 2.28 | 0.000395 | B5U6Y7 | Snaclec CTL-Eoc125 |
| evm.TU.scaffold1986size278736.6 | 1.52 | 0.0004 | Q2EMV9 | Protein mono-ADP-ribosyltransferase PARP14 |
| evm.TU.scaffold9654size34374.2 | 2.5 | 0.000406 | NA | NA |
| evm.TU.scaffold5648size169074.4 | 2.28 | 0.000407 | Q86B61 | Tyramine beta-hydroxylase |
| evm.TU.scaffold2960size121078.2 | 2.35 | 0.000434 | Q8R151 | NFX1-type zinc finger-containing protein 1 |
| evm.TU.scaffold1599size158136.2 | 1.76 | 0.000437 | NA | NA |
| evm.TU.scaffold6904size69075.3 | -2.12 | 0.000446 | NA | NA |
| evm.TU.scaffold2301size281225.14 | 1.74 | 0.000462 | NA | NA |
| evm.TU.scaffold4832size163986.2 | -1.62 | 0.000467 | NA | NA |
| evm.TU.scaffold7254size46811.1 | 1.68 | 0.00048 | NA | NA |
| evm.TU.scaffold8size399332.7 | -1.5 | 0.000488 | NA | NA |
| evm.TU.scaffold588size305867.11 | -1.91 | 0.000499 | NA | NA |
| evm.TU.scaffold8764size38595.1 | 1.68 | 0.000511 | Q2EMV9 | Protein mono-ADP-ribosyltransferase PARP14 |
| evm.TU.scaffold12257size24114.2 | 1.69 | 0.000515 | NA | NA |
| evm.TU.scaffold2905size167108.2 | -2.94 | 0.000518 | P02740 | Serum amyloid A protein |
| evm.TU.scaffold6043size86335.4 | 4.89 | 0.000527 | NA | NA |
| evm.TU.scaffold4938size92729.1 | 2.27 | 0.000529 | NA | NA |
| evm.TU.scaffold588size305867.15 | -1.65 | 0.000561 | NA | NA |
| evm.TU.scaffold1937size287292.5 | -1.79 | 0.000616 | Q03567 | Uncharacterized transporter slc-17.2 |
| evm.TU.scaffold2992size85134.7 | 1.53 | 0.000635 | NA | NA |
| evm.TU.scaffold3547size129194.7 | 1.94 | 0.000644 | NA | NA |
| evm.TU.scaffold16854size10354.1 | -2.47 | 0.000658 | NA | NA |
| evm.TU.scaffold1102size148388.4 | 2.28 | 0.000738 | Q2LD53 | Tryptophan 2,3-dioxygenase |
| evm.TU.scaffold589size246915.11 | -1.73 | 0.000745 | NA | NA |
| evm.TU.scaffold1641size111703.4 | -1.72 | 0.00078 | NA | NA |
| evm.TU.scaffold525size264883.11 | -3.84 | 0.000783 | NA | NA |
| evm.TU.scaffold2520size198016.9 | 1.68 | 0.000797 | NA | NA |
| evm.TU.scaffold3500size108302.6 | -1.95 | 0.000812 | NA | NA |
| evm.TU.scaffold1204size266797.10 | -2.46 | 0.000855 | NA | NA |
| evm.TU.scaffold2971size85384.2 | -1.63 | 0.000869 | NA | NA |
| evm.TU.scaffold10791size60808.8 | 1.57 | 0.00105 | Q2PZL6 | Protocadherin Fat 4 |
| evm.TU.scaffold10243size129472.4 | -3.31 | 0.00109 | NA | NA |
| evm.TU.scaffold1021size251611.4 | 1.8 | 0.0011 | Q28039 | Sodium- and chloride-dependent glycine transporter 1 |
| evm.TU.scaffold2726size89241.4 | -1.51 | 0.00111 | NA | NA |
| evm.TU.scaffold8213size93081.4 | 3.09 | 0.0012 | NA | NA |
| evm.TU.scaffold1043size130741.1 | -1.78 | 0.00122 | NA | NA |
| evm.TU.scaffold494size163391.6 | -2.24 | 0.00127 | Q8IVV2 | Lipoxygenase homology domain-containing protein 1 |
| evm.TU.scaffold1511size382980.30 | 1.64 | 0.00131 | NA | NA |
| evm.TU.scaffold6198size172006.1 | -1.62 | 0.00134 | Q8BZQ2 | Cysteine-rich secretory protein LCCL domain-containing 2 |
| evm.TU.scaffold12636size22904.1 | 1.72 | 0.00155 | NA | NA |
| evm.TU.scaffold879size183110.2 | -1.59 | 0.00159 | NA | NA |
| evm.TU.scaffold1331size227272.13 | -1.69 | 0.00164 | Q68G84 | Phenylalanine aminomutase |
| evm.TU.scaffold1029size217114.8 | -1.78 | 0.00164 | Q8IVV2 | Lipoxygenase homology domain-containing protein 1 |
| evm.TU.scaffold4828size64417.1 | -2.27 | 0.00173 | NA | NA |
| evm.TU.scaffold10155size32176.2 | 2.15 | 0.00177 | Q4G176 | Malonate--CoA ligase ACSF3, mitochondrial |
| evm.TU.scaffold2981size85276.1 | -1.94 | 0.00178 | NA | NA |
| evm.TU.scaffold13516size20036.1 | 1.52 | 0.00179 | NA | NA |
| evm.TU.scaffold4436size159327.6 | 2.29 | 0.00183 | Q9BXJ4 | Complement C1q tumor necrosis factor-related protein 3 |
| evm.TU.scaffold4015size95792.4 | 1.8 | 0.00183 | NA | NA |
| evm.TU.scaffold4691size150772.7 | -1.55 | 0.0019 | H2A0N9 | BPTI/Kunitz domain-containing protein 4 |
| evm.TU.scaffold6407size52220.2 | 1.62 | 0.00214 | Q9W4E2 | Neurobeachin |
| evm.TU.scaffold9171size67839.3 | -2.14 | 0.00219 | NA | NA |
| evm.TU.scaffold8543size75339.5 | 2.46 | 0.00224 | NA | NA |
| evm.TU.scaffold1672size179538.1 | 1.86 | 0.00263 | NA | NA |
| evm.TU.scaffold5241size60644.2 | -1.93 | 0.00266 | NA | NA |
| evm.TU.scaffold1254size464210.7 | 1.54 | 0.00297 | Q9QY40 | Plexin-B3 |
| evm.TU.scaffold7417size86793.2 | 1.52 | 0.00298 | P11717 | Cation-independent mannose-6-phosphate receptor |
| evm.TU.scaffold16280size23477.1 | 1.81 | 0.00309 | NA | NA |
| evm.TU.scaffold10666size29920.1 | -2.89 | 0.00336 | NA | NA |
| evm.TU.scaffold11483size26863.1 | -2.12 | 0.00343 | NA | NA |
| evm.TU.scaffold11021size46776.1 | -1.58 | 0.00349 | NA | NA |
| evm.TU.scaffold8590size39474.1 | 3.2 | 0.00369 | A2RUV4 | Rho GTPase-activating protein 21 |
| evm.TU.scaffold7405size45879.11 | -2.24 | 0.00379 | NA | NA |
| evm.TU.scaffold15684size30752.1 | 1.84 | 0.00392 | NA | NA |
| evm.TU.scaffold8815size134334.6 | 2.27 | 0.00406 | Q53533 | Streptavidin-V2 |
| evm.TU.scaffold3641size124671.3 | 2.59 | 0.00414 | NA | NA |
| evm.TU.scaffold1535size175296.4 | -2.11 | 0.00444 | A3KPQ7 | Cell surface hyaluronidase |
| evm.TU.scaffold10999size50410.1 | -1.8 | 0.00488 | C8YR32 | Lipoxygenase homology domain-containing protein 1 |
| evm.TU.scaffold4353size191616.7 | -1.87 | 0.0051 | NA | NA |
| evm.TU.scaffold5962size55310.2 | 1.64 | 0.00516 | NA | NA |
| evm.TU.scaffold91size419679.8 | -1.59 | 0.0054 | NA | NA |
| evm.TU.scaffold6575size51000.3 | -2.51 | 0.00555 | NA | NA |
| evm.TU.scaffold6562size51107.3 | 1.55 | 0.00565 | NA | NA |
| evm.TU.scaffold462size166051.8 | -1.58 | 0.00569 | NA | NA |
| evm.TU.scaffold5047size126357.10 | -1.57 | 0.00572 | NA | NA |
| evm.TU.scaffold6846size163065.10 | 2.26 | 0.00583 | NA | NA |
| evm.TU.scaffold1082size157023.1 | -1.88 | 0.00601 | NA | NA |
| evm.TU.scaffold4353size191616.5 | -2.04 | 0.00605 | NA | NA |
| evm.TU.scaffold1573size113263.3 | 1.51 | 0.00618 | NA | NA |
| evm.TU.scaffold6715size50122.1 | -1.54 | 0.00642 | NA | NA |
| evm.TU.scaffold5443size69398.3 | -2.19 | 0.00642 | NA | NA |
| evm.TU.scaffold22size297583.2 | 2.25 | 0.00658 | NA | NA |
| evm.TU.scaffold9110size51050.1 | -1.71 | 0.00669 | NA | NA |
| evm.TU.scaffold5944size55442.1 | 1.61 | 0.00671 | NA | NA |
| evm.TU.scaffold1680size293446.11 | -1.96 | 0.00673 | NA | NA |
| evm.TU.scaffold226size262146.12 | -1.92 | 0.00779 | NA | NA |
| evm.TU.scaffold815size266289.2 | -1.6 | 0.00789 | Q3U492 | Kielin/chordin-like protein |
| evm.TU.scaffold11439size82173.6 | 1.82 | 0.00853 | NA | NA |
| evm.TU.scaffold7358size122426.1 | -2.72 | 0.00854 | NA | NA |
| evm.TU.scaffold1188size238585.34 | -1.57 | 0.00866 | NA | NA |
| evm.TU.scaffold4722size240932.5 | 1.68 | 0.0094 | NA | NA |
| evm.TU.scaffold16289size11991.1 | -1.57 | 0.00953 | NA | NA |

**Supplementary Figures**


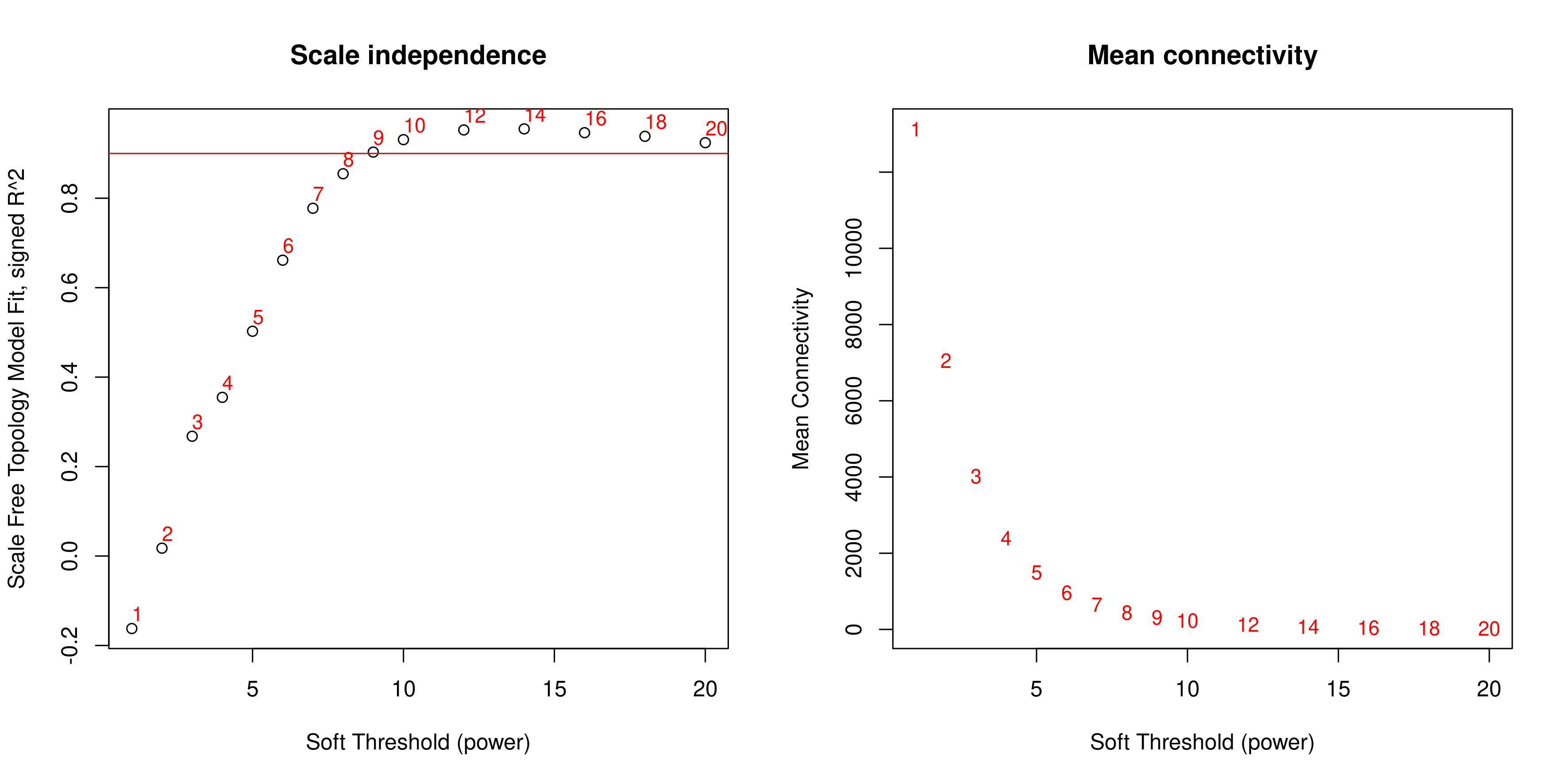


**Figure S1. Determination of the soft-thresholding power (β) in weighted gene co-expression network analysis (WGCNA).** (left) Analysis of the scale-free topology model fitting index (R^2^, y-axis). (right) Mean connectivity for various soft-thresholding powers. The red numbers in the panels denote different soft thresholds. There is a trade-off between maximizing R^2^ and maintaining a high mean number of connections. Thus, we set β = 12.


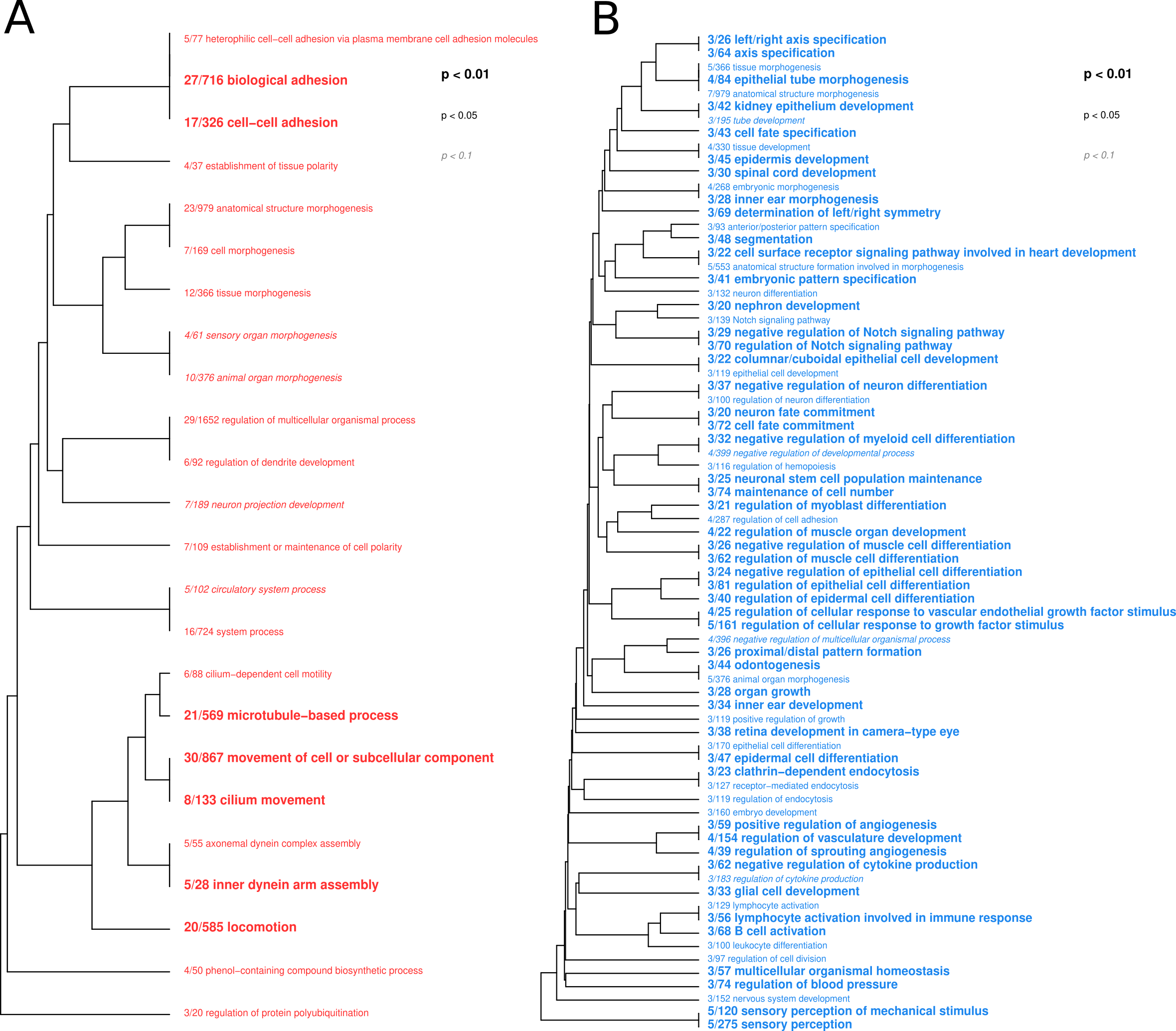


**Figure S2. Gene ontology enrichment for biological processes based on GO_MWU analysis.** (A) GO enrichment for DEGs up-regulated in Fast-growing oysters. (B) GO enrichment for DEGs up-regulated in Slow-growing oysters. The size of the font indicates the significance of the term as indicated by the inset key. The fraction preceding the GO term indicates the number of genes annotated with the term that pass an unadjusted p-value threshold of 0.1. The trees indicate sharing of genes among GO categories (the categories with no separating branch length are subsets of each other).


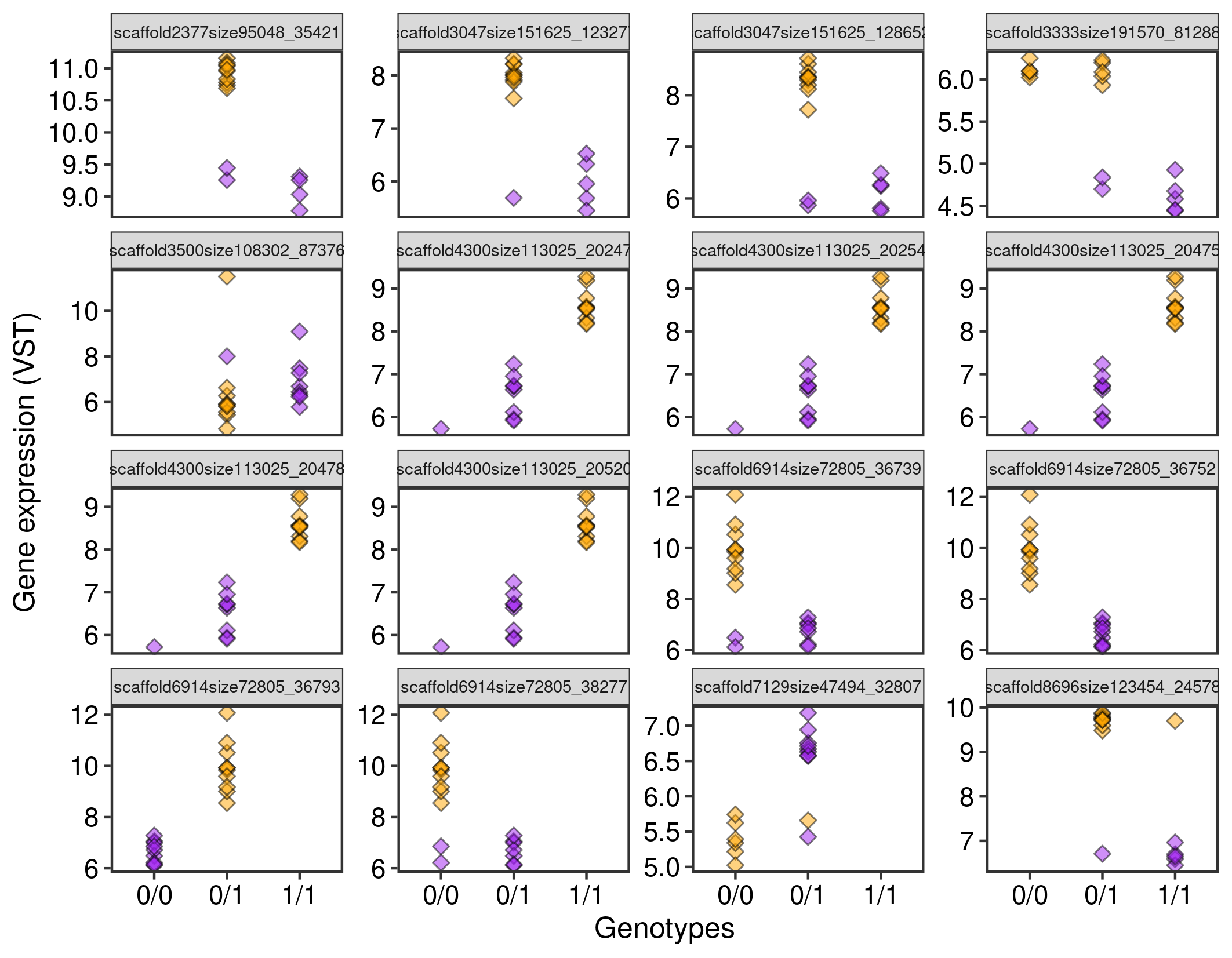


**Figure S3. Relationship between gene expression and genome polymorphism.** Genotype distribution vs. gene expression for the 16 candidate SNPs significantly associated with the growth phenotype (RDA analysis). Colored diamonds represent individuals with orange and purple colors indicating Fast- (F) and Slow- (S) growing phenotypes respectively. Note that for a given SNP, each sample showing miscall genotype is not represented.
